# Contactless and longitudinal monitoring of nocturnal sleep and daytime naps in older men and women: a digital health technology evaluation study

**DOI:** 10.1093/sleep/zsad194

**Published:** 2023-07-20

**Authors:** Kiran K G Ravindran, Ciro della Monica, Giuseppe Atzori, Damion Lambert, Hana Hassanin, Victoria Revell, Derk-Jan Dijk

**Affiliations:** Surrey Sleep Research Centre, University of Surrey, Guildford, UK; UK Dementia Research Institute, Care Research and Technology Centre at Imperial College, London, UK, and University of Surrey, Guildford, UK; Surrey Sleep Research Centre, University of Surrey, Guildford, UK; UK Dementia Research Institute, Care Research and Technology Centre at Imperial College, London, UK, and University of Surrey, Guildford, UK; Surrey Sleep Research Centre, University of Surrey, Guildford, UK; UK Dementia Research Institute, Care Research and Technology Centre at Imperial College, London, UK, and University of Surrey, Guildford, UK; Surrey Sleep Research Centre, University of Surrey, Guildford, UK; UK Dementia Research Institute, Care Research and Technology Centre at Imperial College, London, UK, and University of Surrey, Guildford, UK; UK Dementia Research Institute, Care Research and Technology Centre at Imperial College, London, UK, and University of Surrey, Guildford, UK; Surrey Clinical Research Facility, Faculty of Health and Medical Sciences, School of Biosciences and Medicine, University of Surrey, Guildford, UK; Surrey Sleep Research Centre, University of Surrey, Guildford, UK; Surrey Sleep Research Centre, University of Surrey, Guildford, UK; UK Dementia Research Institute, Care Research and Technology Centre at Imperial College, London, UK, and University of Surrey, Guildford, UK

**Keywords:** contactless sleep technologies, 24-hour sleep assessment, older adults, sleep, nearables, bed sensor, evaluation, actigraphy, home care, sleep diary, Withings Sleep Analyser, Emfit QS, Actiwatch

## Abstract

**Study Objectives:**

To compare the 24-hour sleep assessment capabilities of two contactless sleep technologies (CSTs) to actigraphy in community-dwelling older adults.

**Methods:**

We collected 7–14 days of data at home from 35 older adults (age: 65–83), some with medical conditions, using Withings Sleep Analyser (WSA, *n* = 29), Emfit QS (Emfit, *n* = 17), a standard actigraphy device (Actiwatch Spectrum [AWS, *n* = 34]), and a sleep diary (*n* = 35). We compared nocturnal and daytime sleep measures estimated by the CSTs and actigraphy without sleep diary information (AWS-A) against sleep-diary-assisted actigraphy (AWS|SD).

**Results:**

Compared to sleep diary, both CSTs accurately determined the timing of nocturnal sleep (intraclass correlation [ICC]: going to bed, getting out of bed, time in bed >0.75), whereas the accuracy of AWS-A was much lower. Compared to AWS|SD, the CSTs overestimated nocturnal total sleep time (WSA: +92.71 ± 81.16 minutes; Emfit: +101.47 ± 75.95 minutes) as did AWS-A (+46.95 ± 67.26 minutes). The CSTs overestimated sleep efficiency (WSA: +9.19% ± 14.26%; Emfit: +9.41% ± 11.05%), whereas AWS-A estimate (−2.38% ± 10.06%) was accurate. About 65% (*n* = 23) of participants reported daytime naps either in bed or elsewhere. About 90% in-bed nap periods were accurately determined by WSA while Emfit was less accurate. All three devices estimated 24-hour sleep duration with an error of ≈10% compared to the sleep diary.

**Conclusions:**

CSTs accurately capture the timing of in-bed nocturnal sleep periods without the need for sleep diary information. However, improvements are needed in assessing parameters such as total sleep time, sleep efficiency, and naps before these CSTs can be fully utilized in field settings.

## Graphical Abstract

**Graphical Abstract:**
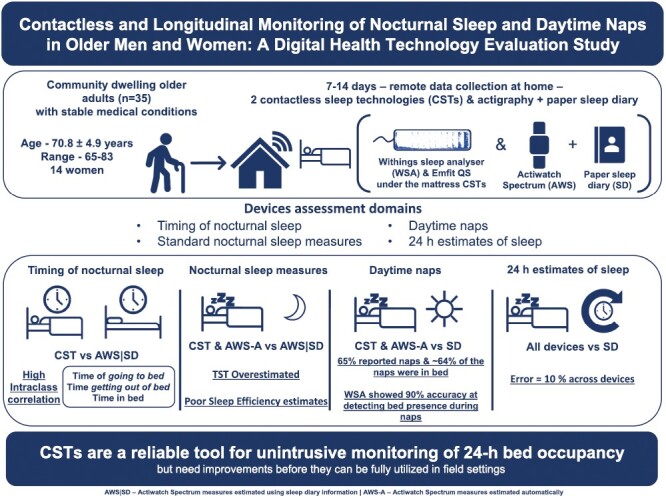

**Statement of Significance**

Contactless sleep technologies that do not pose a burden on participants are promising tools for longitudinal monitoring of sleep in the community. In a comparison with sleep-diary-assisted actigraphy, we show that two under-mattress devices used without sleep diary information provide accurate information on nocturnal sleep timing and 24-hour bed presence. The study population comprised community-dwelling older adults, several of whom had medical conditions such as sleep apnea, arthritis, and type 2 diabetes, which adds to the relevance of these data. With further improvements in their performance, these unobtrusive sleep technologies have significant potential for at-scale and longitudinal monitoring of 24-hour sleep–wake patterns in older adults without the burden of completing sleep diaries.

## Introduction

Basic aspects of the sleep–wake cycle such as very long and very short time in bed (TIB) periods, fragmented sleep, excessive daytime sleepiness, and excessive napping are all associated with negative health outcomes such as accelerated cognitive decline and progression to Alzheimer’s disease (AD) [1–6]. Given the relationship between sleep disruption and health, sleep can be considered as an important noninvasive biomarker for identifying risk and monitoring disease progression. Sleep may also be a potential target for nonpharmacological interventions for slowing AD progression and improving the quality of life in people living with dementia (PLWD) and caregivers [7–9]. To realize the potential of sleep as a biomarker requires robust longitudinal 24-hour objective sleep assessments in community-dwelling older adult populations.

Actigraphy, rest–activity monitoring through wearable devices, in combination with a sleep diary, is currently the most widely used tool for assessing, at-scale, rest–activity patterns as a proxy for sleep–wake patterns [10, 11]. Although actigraphy allows 24-hour objective measurements, it has some drawbacks for longitudinal use in populations such as PLWD. Actigraphy requires individuals to wear a device, most commonly a wrist wearable, and these may not be tolerated by PLWD due to behavioral and psychological symptoms such as irritability and agitation associated with dementia [12–14]. In addition, wearables may need to be regularly recharged and be removed, and then replaced during certain activities such as showers and adherence to these requirements may be problematic for PLWD. Furthermore, for actigraphy to be reliable, it needs to be combined with a daily sleep diary to set the intervals for analysis, the completion of which poses a burden on the participant. Furthermore, sleep diaries may be unreliable, especially in older adults with cognitive impairment [2, 4]. Hence, alternative technologies that are tailored for PLWD are required to overcome the limitations and drawbacks of current longitudinal sleep assessments that rely primarily on actigraphy approaches.

There are many parameters that can be used to quantify the sleep–wake or rest–activity cycle. These include identifying whether a person is asleep or awake, be it at night or during the day, but also whether a person is in bed or not. Quantifying sleep across 24 hours is important because daytime napping can contribute considerably to total sleep time (TST) in PLWDs and may be an indicator of deterioration of the circadian organization of the sleep–wake cycle [2]. Detection of bed presence is also of interest since the TIB which includes the time spent in bed without sleep is an important indicator of decline in physical function and offers additional insight into sleep changes [15, 16].

Environment-embedded contactless sleep technologies (CSTs or Nearables) offer a low-burden approach that is unintrusive and may facilitate long-term, round-the-clock monitoring of sleep-in community-dwelling older adults and PLWD [17]. CSTs, due to their spatially localized nature (eg, under-mattress devices [also known as bed sensors]), have the potential to provide accurate, objective, contextual behavioral information, such as bed presence, alongside sleep-related and physiological (eg, heart rate and respiration rate) measures without the need for sleep diary information for data analysis, unlike actigraphy. Most consumer-grade CSTs are equipped with integration tools and security protocols that allow the devices to be connected to home networks and stream the data continuously to secure cloud storage platforms. Hence, CSTs have the potential to allow the creation of digital health platforms to collect and analyze sleep, vital signs, and behavioral information round-the-clock longitudinally, at scale in community-dwelling populations.

Most CSTs use a ballistography signal as the basis for their quantification of the sleep–wake cycle. However, the signal acquisition technology employed, proprietary data handling, feature extraction, and sleep-stage detection pipelines vary across CSTs. Many sleep-stage detection pipelines will have been developed based on data obtained in healthy people under controlled laboratory conditions. Some studies have evaluated individual CSTs in older adults, but these studies have been limited to either lab or home settings with assessment limited to simple bed presence or sleep summary assessments of nocturnal sleep only [18–24].

To the best of our knowledge, there is no existing research comparing multiple CSTs to the current standard of longitudinal sleep assessment at home, that is, actigraphy combined with sleep diary information, across multiple sleep monitoring domains in older adults [15, 16, 18–22]. The primary aim of this study is to evaluate the 24-hour sleep assessment capability of two under-mattress CSTs and automated actigraphy analysis in comparison to sleep-diary-assisted actigraphy at home in a population of community-dwelling older adults with a health status that is somewhat representative of the older population.

## Methods

### Cohort characteristics and data collection procedure

Thirty-five community-dwelling older adults were recruited for the study. The participants were aged 65 years and over (range: 65–83 years; mean ± standard deviation [*SD*]: 70.8 ± 4.9; 21 men: 14 women), lived independently, and could carry out their normal daily activities. Potential participants were identified through the Surrey Clinical Research Facility (CRF) recruitment database and potential eligibility was first assessed via a telephone screen. Participants who passed the telephone screen attended a screening visit at the CRF where eligibility was determined. The presence of medical conditions such as hypertension, type 2 diabetes, and arthritis was not an exclusion criterion provided that their medical condition has been stable with no recent hospitalization and no anticipated change to current treatment or initiation of new therapy during the study. Eligible participants had to be able to comply with study procedures and no safety concerns posed to their health by participating in the study. Participants with self-declared stable, controlled physical health conditions and no history of neurological or mental health problems among other standard criteria (nonsmoker; consumed <28 units of alcohol per week; no substance use; absence of self-reported sleep disorders such as REM sleep behavior disorder, etc.) were considered eligible for enrollment to the study. The study was conducted in line with the Declaration of Helsinki and the Principles of Good Clinical Practise, and the study protocol obtained a favorable opinion from the University of Surrey ethics committee. All participants provided written informed consent before any study procedures were performed.

The data were collected in two cohorts (cohort 1—18 participants [January to March 2020]; cohort 2—17 participants [June to November 2021]). In both cohorts, we first collected data at home and then in a sleep laboratory setting including a full clinical polysomnography (PSG) conducted according to American Academy of Sleep Medicine (AASM) guidelines. Here we focus on data collected at home for 7–14 days. The participants were trained on wearing the actiwatch, using the sleep monitoring devices (two under-mattress CSTs), and completing the consensus sleep diary [25]. In cohort 2, an extended version of the sleep diary was used to collect additional information on the location and timing of daytime naps as well as nocturnal bed exits. The sleep diary was collected on paper, and the participants completed questions about their nocturnal sleep episode upon awakening and, at the end of the day, completed questions relating to daytime events including naps and alcohol and caffeine consumption. The sleep diary lights-off period information was derived from the questions on the timing of lights off (*What time did you try to go to sleep?*) and lights on (*What time was your final awakening?*) while the TIB information was estimated using timing of *going to bed* (*What time did you get into bed?*) and *getting out of bed* (*What time did you get out of bed for the day?*) questions.

The participants were instructed to wear the Actiwatch Spectrum (AWS) continuously on their dominant wrist to allow for the quantification of 24-hour activity and rest behavior. They were instructed to carry out their normal day-to-day activities and were asked to only remove the AWS if it was going to get wet. At the end of the study, the participants were asked to fill out a device acceptability questionnaire to understand the comfort, ease of use, and any problems faced while handling the different devices at home.

### Actigraphy data

The AWS (Philips Respironics) was used as the reference device, in combination with the sleep diary, for the at-home evaluation of the sleep summary estimation performance of CSTs. The AWS was configured to collect and output activity and sleep–wake labels at 1-minute epochs for 7–14 days of home recording. The device locally stores the collected data that were subsequently downloaded and analyzed using the Philips Actiware software version 6.0.7. The AWS was synchronized to the local clock time by Actiware during configuration. The AWS provided both sleep summary estimates and epoch-by-epoch (EBE) sleep–wake estimates. The EBE estimates are derived by the AWS based on the activity threshold set by the user (to determine inactivity and discriminate sleep and wake states), and this affects the sleep summary estimate output. We investigated the effect of different sensitivity settings by comparing the AWS scoring against gold-standard PSG in cohort 1. The medium threshold (activity count = 40) setting of the AWS was found to be optimal. See Supplementary Materials for the results of the evaluation of the effect of threshold.

The AWS summary measures were generated by using two different analysis periods: the analysis period automatically determined by the Actiware 6.0.7 software (AWS-A) and analysis period set manually using the sleep diary lights-off and lights-on information. The manual analysis data, that is, AWS-given sleep diary information (AWS|SD), were used as the primary reference sleep summary estimate for evaluating the CSTs since this approach is recommended by the AASM [26].

The bed presence information collected by the sleep diary was inspected manually by an examiner using the AWS light and activity data to identify outliers in the *going to bed* and *getting out of bed* data used in the bed presence analysis [19]. This approach involved comparing the sleep diary bed presence information and the concurrent changes in activity and light information from the AWS to verify the sleep diary entries. Any sleep diary bed presence information entry deviating 30 minutes or over from the AWS data was adjusted to the time of concurrent AWS activity and light information change. In the absence of sleep diary information, the bed presence information was considered as unavailable.

The AWS information related to daytime naps was obtained by running an automated AWS analysis at medium threshold in the Actiware software set to detect both major and minor sleep intervals. The major intervals correspond to the nocturnal sleep periods and the minor intervals correspond to potential nap periods automatically detected by AWS.

### Contactless sleep technologies

The two CSTs evaluated in this study were the WSA (Withings Sleep Analyser, Withings, France) and Emfit (Emfit QS, Emfit Ltd, Finland), which are both contactless under-mattress devices designed for use by only one individual and use ballistographic sensing to collect activity and physiological information for sleep monitoring. The WSA employs a pneumatic sensing unit while the Emfit uses an electromechanical film sensor for acquiring the ballistographic signal. The devices were enabled throughout the home study period and uploaded data via a Wi-Fi hotspot to secure cloud servers. Both the devices generate data only when the bed is occupied and hence the presence of data recording acts as a source of 24-hour bed presence information. Sleep was automatically scored by the devices using proprietary algorithms. Both devices generate summary statistics for each recording period. In addition, EBE sleep-stage classification data can also be accessed. The WSA provides the EBE estimates at 1-minute intervals while the temporal resolution of the output from the Emfit is at 30 seconds. The detected “sleep” stages are wake, rapid eye movement (REM) sleep, light sleep (assumed to be comparable to N1 or N2 sleep), and deep sleep (assumed to be comparable to N3 sleep).

### Data overview—curation, availability, and missing data

The CST devices used in the study were connected to the internet using the TP-Link M7350 4G LTE Mobile Wi-Fi router (TP-Link UK Ltd) and were synchronized to the respective network times and collected the sleep data simultaneously. The daylight-saving correction was applied to the UTC time series of the devices to allow data analysis to be performed relative to local clock time. The CST data collected over the entire study period were available for download from the application programming interface for WSA and a web interface for Emfit. The sleep summary and EBE data were downloaded as .json files for WSA and .csv for Emfit. The AWS data were downloaded and processed using the Actiware software and exported as .csv files. For each of the devices, the data export and labeling were performed and checked by a single individual followed by a sanity check of the recording periods programmatically using sleep diary information.

Data collected from the CSTs and AWS were date matched with the sleep diary information. This was followed by identification and elimination of outliers and creation of clean summary and time-series data tables for all the devices. The summary measures were considered as data outliers if the recorded value were outside the 99th percentile. One participant’s data were lost for AWS in cohort 2. The data import, preparation, and analysis were performed in MATLAB 2022a.

#### Bed presence.

Both WSA and Emfit provide EBE information on bed presence, and these data were used to identify the major nocturnal in-bed period and create the CST times for *going to bed* and *getting out of bed*. For AWS-A, we used the major interval start and end times.

#### Nap information.

The sleep diary nap information provided by the participants was considered as the ground truth nap information. It should be noted that participants may have forgotten to record the nap information contributing to loss of ground truth data due to human error.

The device nap periods were derived from summaries automatically generated by each of the compared devices. For AWS-A, the naps correspond to the minor sleep intervals outside the major nocturnal sleep periods detected by Actiware based on inactivity information, while for the CSTs, the nap periods correspond to daytime in-bed periods automatically identified by the respective device.

We identified that the WSA did not generate automatic summaries for those periods during which the device was activated (ie, bed presence was detected) but the in-bed period did not contain sleep epochs. For these in-bed periods, the time series of EBE “sleep scoring” was available. To accurately determine the device capabilities, the nap analysis was conducted in two ways: (1) based on the available summary files and (2) using the EBE bed presence information from WSA. The Emfit does not contain EBE information (in the download data) on the bed presence less than 2 hours and hence could not be used in this analysis.

## Data Analysis

### Sleep summary agreement assessment

For the sleep summary agreement assessment, we compared bed presence estimation, all-night sleep summary measures, daytime naps, and 24-hour sleep summary measures. Here, the sleep summary measures include standard nocturnal sleep measures: TST, sleep onset latency (SOL), wake after sleep onset (WASO), and sleep efficiency (SEFF).

AWS|SD data were considered the primary reference for the summary analysis, and for the nights for which we did not have sleep diary information, the comparison of the CST to the AWS|SD was not made. The bed presence and nap analysis were performed against data common between sleep diary and devices (see Table 1). The *going to bed* and *getting out of bed* times from the sleep diary (corrected using AWS activity and light data) were used for the bed presence agreement analysis which involves *going to bed* and *getting out of bed* times and TIB duration. The assessment of the agreement between devices of all-night measures was conducted on estimates based on the analysis period automatically determined by the device (AP-A) and analysis period manual set using the sleep diary information (AP-SD).

**Table 1.** Demographical and clinical characteristics of participants

Characteristics	Cohort 1	Cohort 2	Total
*N*	18	17	35
Age (years)	69.67 (5.04) [65, 80]	72 (4.49) [65, 83]	70.8 (4.86) [65, 83]
Women (*n* [%])	8 [44.44]	6 [35.29]	14 [40]
Height (cm)	167.82 (9.36) [153, 183]	169.76 (10.12) [153, 191]	168.76 (9.64) [153, 191]
Weight (kg)	76 (17.64) [59, 121]	75.48 (18.87) [48.6, 134.4]	75.75 (17.98) [48.6, 134.4]
BMI (kg/m^2^)	27.03 (4.84) [21.87, 39.75]	26.42 (4.71) [20.00, 36.8]	26.73 (4.72) [20.00, 39.75]
AHI (events/hour)	20.89 (17.46) [1.60, 66.7]	18.96 (13.62) [4.20, 58.8]	19.95 (15.51) [1.60, 66.7]
Surgical Procedures (*n* [%])	2 [11.11]	8 [47.06]	10 [28.57]
Comorbidities (*n* [%])			
Obesity (BMI > 30)	3 [16.67]	3 [17.65]	6 [17.14]
Type 2 **d**iabetes	1 [5.56]	1 [5.89]	2 [5.71]
Arthritis	4 [22.22]	2 [11.76]	6 [17.14]
Hypertension	2 [11.11]	0	2 [5.71]
Acid reflex	1 [5.56]	1 [5.89]	2 [5.71]
Mild sleep apnea (5 > AHI < 15)	8 [44.44]	8 [47.06]	16 [45.71]
Moderate sleep apnea (15 ≤ AHI < 30)	4 [22.22]	5 [29.41]	9 [25.71]
Severe sleep apnea (AHI ≥ 30)	5 [27.78]	3 [17.65]	8 [22.85]
Cognition, sleep, and functional status assessment			
Mini-Mental State Examination (MMSE)	28.44 (1.46) [25, 30]	28.88 (1.32) [25, 30]	28.66 (1.39) [25, 30]
Pittsburgh Sleep Quality Index (PSQI)	4.22 (1.86) [1, 7]	4 (2.35) [1, 10]	4.11 (2.08) [1, 10]
Epworth Sleepiness Scale (ESS)	3.72 (2.67) [1, 9]	3.47 (2.32) [0, 8]	3.60 (2.47) [0, 9]
International Consultation on Incontinence Questionnaire (ICIQ)	1.06 (1.86) [0, 6]	1 (1.66) [0, 4]	1.03 (1.74) [0, 6]
Instrumental Activities of Daily Living (ADL)	7.94 (0.24) [7, 8]	7.94 (0.25) [7, 8]	7.94 (0.24) [7, 8]

The values shown are the mean followed by the (standard deviation) and range [minimum, maximum].

The daytime nap concordance analysis was performed using the daytime automatic summaries generated by all the devices as well as the EBE bed presence information available from WSA (WSA-BP). Both the incidence of naps and duration of the naps were compared to the sleep diary ground truth nap information for both the approaches. To facilitate accurate nap analysis across all compared devices (AWS-A, WSA-A, WSA-BP, and Emfit-A), we eliminated naps detected in the 2-hour period prior to the time of *going to bed* time and after the time of *getting out of bed* time to avoid the effect of sleep transition period as recommended by Peng Li et al. [2]. Following this elimination process, naps with durations shorter than 10 minutes or longer than 300 minutes were excluded. Combined, the above two nap filtering approaches remove outlier nap periods from the collected data. For the purposes of evaluation in this study, any device nap period that did not overlap with the sleep diary ground truth nap information was considered a “false” nap. These devices detected “false” naps are referred to as *unreported naps* (ie, not reported in the diary) in the rest of the manuscript.

One participant’s data could not be used across all compared devices due to missing AWS data. The common days of data available between AWS|SD and each of the devices are summarized in Table 1. The common days were similar for the 24-hour agreement estimation since the AP-A estimates of the devices are compared against sleep diary. The 24-hour TST was computed as the sum of all-night TST estimate and daytime TIB of the nap episodes. In the ideal case, it should be the sum of the nocturnal TST and daytime TST. However, nap TST and other sleep measures were often not available from the sleep diary. The error introduced by this is likely to be small because nap duration contributes to a small percentage of the 24-hour estimate compared to nocturnal TST. For WSA, both the automatically generated naps episodes (WSA-A) and the nap episodes derived from the bed presence information (WSA-BP) were used to estimate the 24-hour TST.

For all sleep summary agreement analyses, Bland Altman measures acted as the primary tool for comparison. Bias, limits of agreement (LoA), and minimum detectable change (MDC) are reported with 95% confidence intervals, where applicable. MDC is the smallest change in the estimate that can be detected by the device that exceeds the measurement error. Due to the large sample size of the estimates, the normality of the differences was evaluated visually using QQ-plots and residual plots. Logarithmic transformation was performed to correct the deviation from normality where applicable. Furthermore, we also corrected for the presence of proportional bias (condition where the linear regression reveals nonzero slope of bias line) and homoscedasticity of the residuals (nonconstant variance) [27–29]. In the presence of proportional bias, the bias is represented as


$$\text{Bias}=a_{0}+a_{1}X$$

Here, $a_{1}a_{1}$ is the slope and $a_{0}a_{0}$ is the intercept of the linear regression between the *X* (ie, the average between the device and reference (AWS|SD) estimate) and *Y*-axis of the Bland Altman analysis. In case of deviation from homoscedasticity, the bias is represented as


$$\text{AR}=b_{0}+b_{1}X$$

Here, *b*_1_ is the slope and *b*_0_ is the intercept of the linear regression between the *X*-axis of the Bland Altman analysis and absolute of residuals (AR) of the test for proportional bias. Under this assumption, the 95% LoA are given by


$$\text{LoA}=\text{Bias}\pm 2.46\left(b_{0}+b_{1}X\right)$$

Other agreement measures include absolute intraclass correlation (ICC) with two-way random effects (ie, case of single measurements), symmetric mean absolute percentage error (SMAPE), and standardized absolute difference (SAD).

Since the data per participant were collected over 7–14 days, the correct ICC computational case is repeated measurements with nonconstant reference value (day-to-day variability in reference value). This requires a large number of observations since the 95% confidence intervals can only be computed via bootstrapping. Due to the small sample size in our case, we have computed the ICC per participant assuming the case of single measurements. The ICC is reported as the mean and 95% confidence interval of individual participant ICC.

### Epoch-by-epoch concordance assessment

EBE concordance analysis was carried out over the sleep diary lights-off period and for all in-bed periods between 18:00 on 1 day and 12:00 hours the following day. To facilitate accurate EBE concordance assessment, the sleep–wake time series of AWS and the CSTs were aligned by estimating the cross-correlation function and determining the lag within a 10-minute window that provides the highest agreement. The concordance analysis was performed at the device resolution, that is, 60-second intervals in case of WSA and 30 seconds in case of Emfit. The 60-second AWS sleep–wake time series were imputed with the adjacent minute label to derive hypnogram at 30-second epoch resolution. Sensitivity Specificity, Accuracy, Matthew’s correlation coefficient (Matthew’s CC), and F1-score were used to evaluate the EBE concordance of the CSTs. The Matthew’s CC is a reliable statistical measure of performance that accounts for the class imbalances in the data. The Matthew’s CC ranges between −1 (worst) and +1 (best) and is represented as


$$\text{Matthew's CC=}\frac{\text{True positive}\times \text{True negative}-\text{False positive}\times \text{False negative}}{\sqrt{\left(\text{True positive}+\text{False positive}\right)\left(\text{True positive}+\text{False negative}\right)\left(\text{True negative}+\text{False positive}\right)\left(\text{True negative}+\text{False positive}\right)}}$$

## Results

### Study population characteristics

The age range of the participants in the study was between 65 (youngest) and 83 (oldest) years and 57% of the participants reported comorbidities such as arthritis, type 2 diabetes, hypertension, obesity, etc., and corresponding medications in their health questionnaire (see Table 1). About 29% (*n* = 10) of the participants had a medical history of surgical procedures. The Mini-Mental State Examination (MMSE) scores of all the participants were above the cutoff (23) for clinically significant cognitive impairment. None of the participants had significant urinary incontinence (maximum ICIQ score: 6) and all were able to perform activities of daily living independently (minimum ADL score: 7). About 69% of the participants did not have significant sleep disturbance according to the Pittsburgh sleep quality index (*n* = 24, PSQI < 5), and were not excessively sleepy as indicated by the screening Epworth Sleepiness Scale (mean ESS: 3.6). The clinical PSG performed during the lab visit following the home data collection, however, revealed that 48.6% of the participants had severe (*n* = 8, apnea–hypopnea index [AHI]: >30) or moderate (*n* = 9, AHI: 15 to <30) sleep apnea while 45.7% (*n* = 16) of the participants had mild apnea (AHI: 5 to <15).

### Overview of the at-home data

In total, 401 days of data were collected at home with 9.17 ± 0.71 (mean ± *SD*) days of data in cohort 1 (18 participants) and 13.88 ± 0.33 days in cohort 2 (17 participants; see Table 2). The participants reported that the WSA and Emfit were very easy to use. Although the AWS was also easy to use, the participants reported lower comfort using the AWS compared to the CSTs.

**Table 2.** Data availability for devices used in the study

Devices	Participants (*N* = 35)	Total Days of data collected (401 days)	Days available for bed presence analysis	AWS|SD (days)
Sleep diary	35	392	—	-
AWS	34	387	385	379
WSA	27	321	309	306
Emfit	16	228	210	209

**Figure 1.**
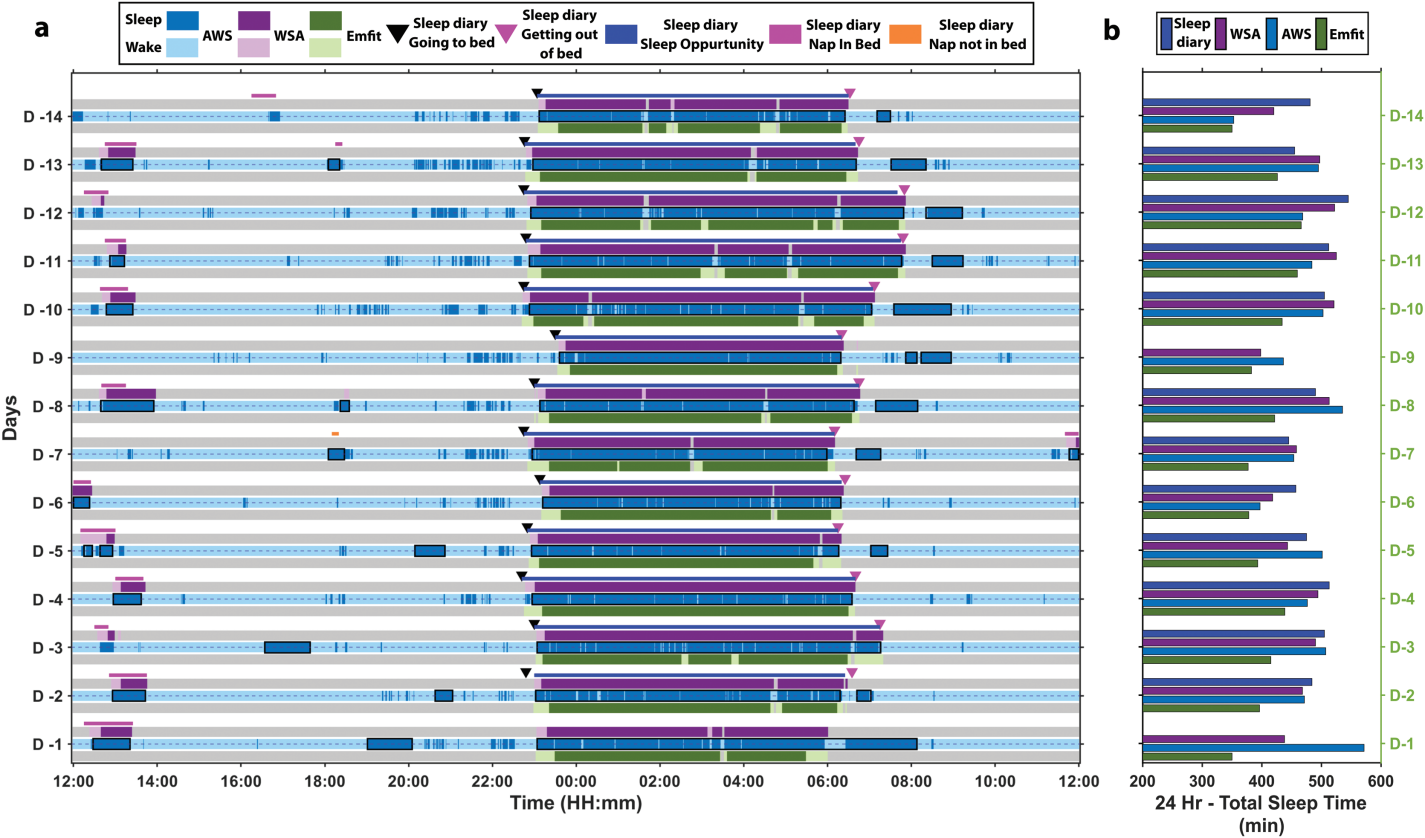
Sleep behavior patterns over 14 days (D-14 to D-1) at home in a male participant aged 72. (A) The raw epoch-by-epoch sleep–wake time-series data and the associated sleep diary information. (B) The 24-hour total sleep time estimates for the four methodologies used. See Results for more detailed explanation.

As the first step of the evaluation process, we visualized the data collected by the sleep diary, AWS, and CSTs. This allowed us to identify data outliers, unique participants, and to structure and organize our analysis that best suited the collected data. An example of data collected at home for one of the participants with consistent daytime naps along with the 24-hour TST estimate is depicted in Figure 1. The colored regions in the figure denote data presence while the gray regions denote the absence of data. For all devices, the darker colored regions in Figure 1 denote sleep while the lighter regions denote wake. The AWS determines sleep–wake status with a 1-minute resolution and then applies a proprietary sleep summary generation algorithm to the sleep–wake time series to determine major and minor sleep intervals. The regions highlighted in the figure by the black boxes in AWS data indicate sleep episodes automatically detected by this Actiware algorithm. Please note that many short daytime “sleep” episodes are not classified as minor sleep intervals. The sleep diary information on timing of *going to bed* and *getting out of bed*, sleep opportunity and naps are also represented in the figure. In this example, both WSA and Emfit and AWS-A detect the nocturnal *going to bed* and *getting out of bed* times accurately and are in close agreement with the sleep diary information.

In this participant, WSA and AWS reliably detected sleep-diary-reported nap periods in particular those between 12:00 and 14:00 (see Figure 1). AWS detected numerous additional sleep periods (minor sleep intervals) outside the major nocturnal episode. These occurred primarily in the morning shortly after the major nocturnal sleep period. The nap periods immediately (within a 2-hour window) following and before the major nocturnal episodes were eliminated in the nap analysis as discussed in the data curation section. The 24-hour sleep estimates depicted on the right panel of Figure 1 show that all devices to some extent, but consistently, overestimated the 24-hour TST compared to the sleep diary.

Supplementary Figure 9 depicts the discrepancy between the daytime bed occupancy detected by the CSTs and the automated summaries. The automatic summaries detected by the devices are superimposed on the EBE data available from compared devices. Here, the WSA accurately detected bed presence for all naps in bed reported in the sleep diary but did not generate automated summaries for bed presence periods determined to be wake bouts by the device algorithm. This showcases the influence of the device sleep detection algorithm on the automated summaries generated. Emfit, on the other hand, did not have EBE data outside the nocturnal sleep periods but generated summaries that coincided with the sleep-diary-reported naps.

### Nocturnal bed presence estimation

**Figure 2.**
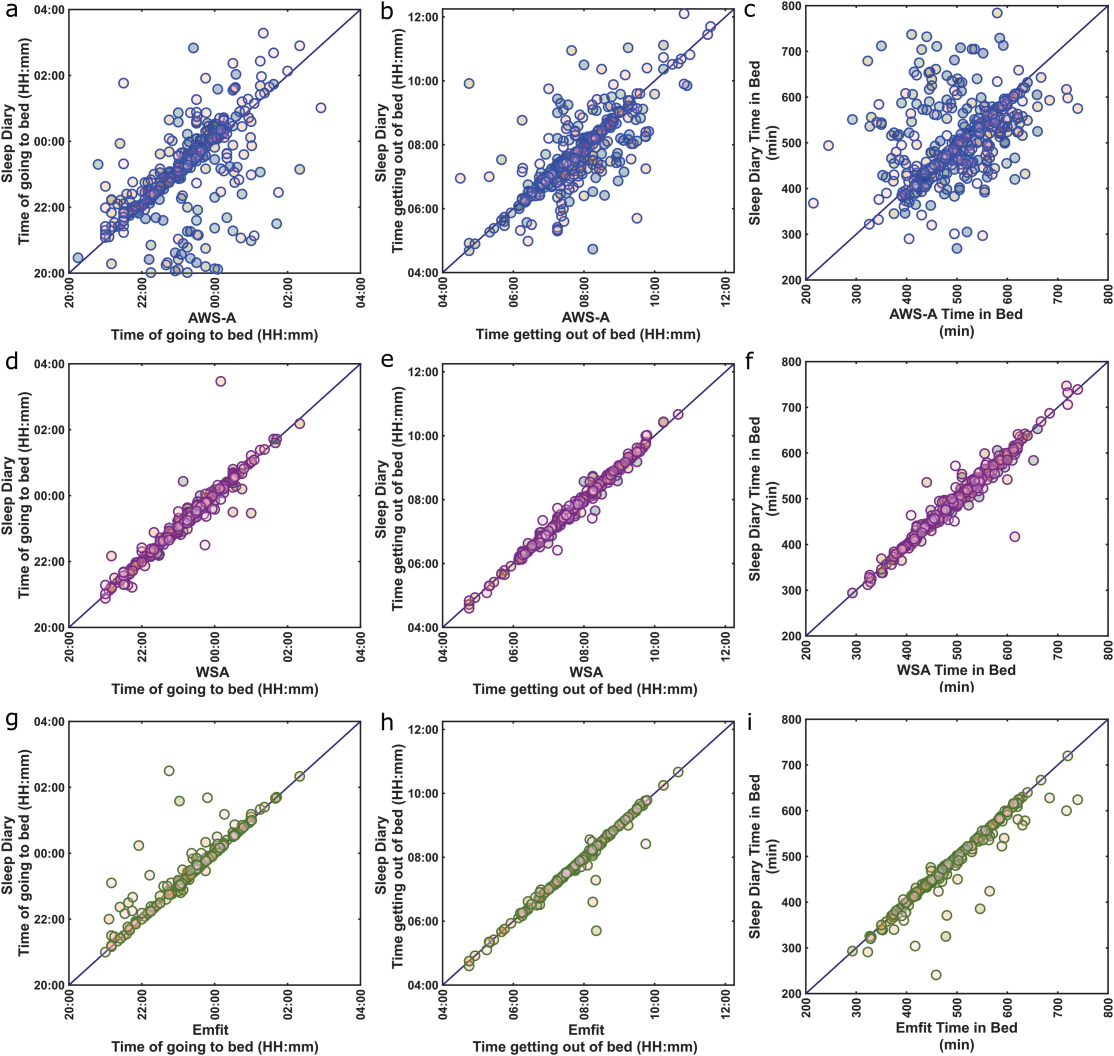
Comparison of timings of bed entries and exits for the Actiwatch, Withings Sleep Analyser, and Emfit. Scatterplots represent the agreement between the device and the sleep diary estimates. AWS-A represents the automatic Actiwatch analysis. The number of nights is 379 for AWS-A, 306 for WSA and 205 for Emfit.

For bed presence analysis, the sleep diary *going to bed* and *getting out of bed* times were considered as the reference estimate. This information corresponds to the times the participant got into bed and got out of bed at the beginning and end of the major nocturnal sleep episodes, respectively. A total of 28 *going to bed* and 9 *getting out* of bed sleep diary entries were corrected as described in the methods. The scatter plots depicting the *getting out of bed* and *going to bed* concordance of the CSTs with sleep diary and the distribution of the *going to bed* and *getting out of bed* times as recorded by the sleep diary, WSA, and Emfit are shown in Figure 2 and Supplementary Figure 1. The agreement metrics for the estimates are given in Table 3.

**Table 3.** Bed presence agreement metrics

Bed presence measure		Bias [95% CI]	*p*-Value	LoA lower bound [95% CI]	LoA upper bound [95% CI]	MDC	SAD [95% CI]	SMAPE [95% CI]	ICC [95% CI]
Time of going to bed	AWS-A	−22.82 (98.16) [−32.73, −12.91]	<.001	−215.22 [−232.17, −198.26]	169.57 [152.62, 186.53]	192.39	0.67 [0.57, 0.77]	38.27 [34.43, 42.11]	0.34 [0.26, 0.43]
	WSA	2.06 (16.74) [0.17, 3.94]	.032	−30.75 [−33.97, −27.52]	34.86 [31.64, 38.08]	32.80	0.12 [0.01, 0.23]	13.06 [10.29, 15.83]	0.90 [0.86, 0.94]
	Emfit	8.13 (25.83) [4.61, 11.66]	<.001	−42.50 [−48.53, −36.47]	58.76 [52.73, 64.79]	50.63	0.15 [0.01, 0.28]	10.99 [7.79, 14.19]	0.78 [0.68, 0.88]
Time getting out of bed	AWS-A	−7.78 (52.32) [−13.06, −2.49]	.004	−110.31 [−119.35, −101.28]	94.76 [85.72, 103.8]	102.54	0.41 [0.31, 0.51]	3.31 [2.77, 3.84]	0.58 [0.5, 0.66]
	WSA	0.12 (8.20) [-0.8, 1.04]	.80	−15.96 [−17.54, −14.38]	16.19 [14.61, 17.77]	16.07	0.07 [−0.04, 0.18]	0.50 [0.41, 0.59]	0.93 [0.90, 0.97]
	Emfit	−2.57 (15.09) [−4.63, −0.52]	.014	−32.14 [−35.66, −28.62]	26.99 [23.47, 30.52]	29.57	0.06 [−0.08, 0.19]	0.42 [0.18, 0.65]	0.93 [0.89, 0.98]
Time in bed (TIB)	AWS-A	15.04 (111.7) [3.76, 26.33]	.009	−203.89 [−223.18, −184.59]	233.98 [214.68, 253.27]	218.93	0.79 [0.69, 0.89]	6.97 [6.19, 7.76]	0.4 [0.33, 0.46]
	WSA	−1.93 (18.32) [−3.99, 0.13]	.066	−37.83 [−41.36, −34.31]	33.97 [30.45, 37.50]	35.90	0.12 [0.01, 0.23]	0.94 [0.77, 1.12]	0.92 [0.89, 0.95]
	Emfit-A	−10.65 (29.57) [−14.68, −6.62]	<.001	−68.62 [−75.52, −61.71]	47.31 [40.41, 54.22]	57.97	0.14 [0.01, 0.28]	1.30 [0.83, 1.76]	0.84 [0.78, 0.91]

Metrics of agreement for the timing of *going to bed*, *getting out of bed* and the duration of time in bed (TIB). The Emfit estimates are heteroscedastic, and the corrected Limits of Agreement (LoA) are depicted as given in Equation (2) as Bias ± 2.46 (7.69 + 21.53 [Average]); *b*_1_ = [2.53, 12.86]; *b*_0_ = [16.37, 26.68]. The values shown are the mean followed by the (standard deviation) and [95% confidence interval]. The metrics include Bias—difference in measurement between the Device and Sleep diary (Device—Sleep diary); *p*-value indicating the significant difference in the metric estimation tested using paired *t*-test; lower and upper bounds of the Bias; minimum detectable change (MDC)—smallest detectable change independent of measurement error (half of Bland–Altman agreement width); standardized absolute difference (SAD)—directionless version of Cohen’s *d*; symmetric mean absolute percentage error (SMAPE)—mean error in measurement; absolute intraclass correlation with two-way random effects (ICC)—measures of measurement reliability. The ICC estimates were computed for each participant, and the mean and 95% confidence interval are reported. The number of participants (days) used in each of the devices: WSA—27 (306) and Emfit—16 (205). All the values in the table are rounded to two decimal places.

The earliest and latest *going to bed* times reported by the participants were 20:15 and 02:55, respectively, while for the *getting out of bed*, the earliest and latest times were 04:30 and 11:20. The mean TIB was 8 hours 22 minutes (Sleep diary: 78.5 minutes) while the shortest and longest TIB were 3 hours 35 minutes and 12 hours 20 minutes, respectively. The mean participant *going to bed* times ([HH:mm] ± *SD* [minutes]) as recorded by the sleep diary, AWS-A, WSA, and Emfit were 23:04 ± 54, 22:39 ± 91 23:08 ± 53, and 23:14 ± 53 while the *getting out of bed* times were 07:34 ± 67, 07:26 ± 77, 07:30 ± 63, and 07:25 ± 64.

Overall, the correspondence between the sleep diary estimates and the estimates from the CSTs was good while AWS-A had poor correspondence (see Figure 2 and Table 3). The WSA (Bias: 2.06 minutes, ICC: 0.9) and Emfit (Bias: 8.13 minutes, ICC: 0.78) bed entries were consistently earlier than the *going to bed* while the AWS-A (Bias: −22.82 minutes, ICC: 0.34) bed entry was later. The *getting out of bed* estimate of WSA (Bias: 0.12 minutes, ICC: 0.93) was very close to the estimate and did not show significant differences while the AWS-A (Bias: −7.78 minutes, ICC: 0.58) and Emfit (Bias: −2.57 minutes, ICC: 0.93) estimates of time *getting out of bed* were earlier than the time. The AWS-A (Bias: 15.04 minutes, ICC:0 .4) overestimated the TIB while both WSA (Bias: −1.93 minutes, ICC: 0.92) and Emfit (Bias: −10.65 minutes, ICC: 0.84) underestimated the derived TIB estimate. The other agreement metrics showed a similar outcome, that is, both CSTs were good at estimating these sleep parameters with the WSA performing slightly better at estimating bed presence compared to Emfit.

### All-night sleep summary measures

The participant sleep-diary-reported TST ranged between 120 and 605 minutes (mean ± *SD*: 405.96 ± 84.00) while the WASO ranged between 0 and 300 minutes (mean ± *SD*: 28.22 ± 37.84) leading to sleep efficiencies ranging from 40.00% to 98.81% (mean ± *SD*: 88.09 ± 11.19). The reported SOL ranged between 0 and 120 minutes (mean ± *SD*: 19.49 ± 19.9).

**Figure 3.**
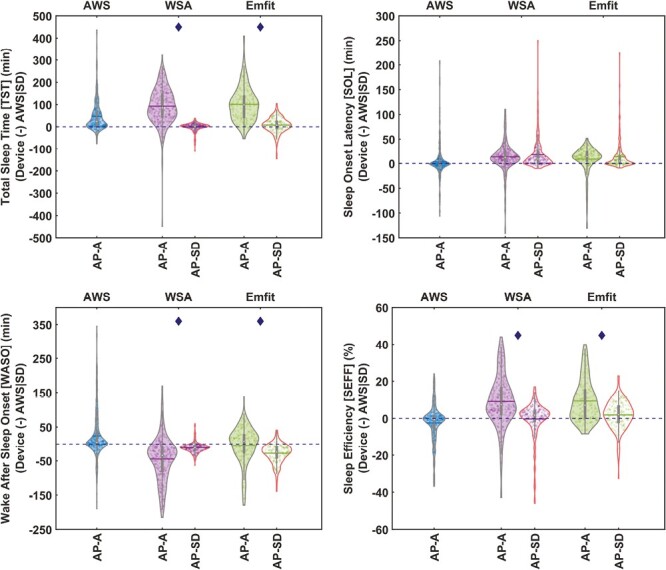
Differences in the all-night sleep summary measure estimations of CSTs and AWS devices against AWS|SD (Device (-) AWS|SD). The violins with gray outline show the device estimates based on Analysis period—Automatic (AP-A, analysis period determined by the device), and the violins with red outline depict the Analysis period set with the aid of sleep diary information (AP-SD, analysis period set from sleep diary lights off to lights on). The number of participants (days) used for each of the devices is AWS—34 (379), WSA—27 (306), and Emfit—16 (205).

The all-night sleep summary measures common across all the compared devices (TST, SOL, WASO, and SEFF) were estimated for both AP-A and AP-SD and compared against the AWS|SD reference. The differences between the device and the AWS|SD measures (ie, Bias) are presented in Figure 3 while the agreement metrics are reported in Table 4 for the AP-A estimates and the corresponding scatter plots of the estimates are depicted in Supplementary Figure 2. In Figure 3, the horizontal blue dotted line indicates no differences compared to AWS|SD. The data points above the line indicate overestimations while points below the line indicate underestimations. The extended Bland–Altman measures correcting for proportional bias and deviation from homoscedasticity are provided in Supplementary Figure 3 and Supplementary Table 1.

**Table 4.** All-night sleep–wake summary measure agreement metrics (AP-A estimates compared to AWS|SD)

Sleep measure		Bias [95% CI]	*p*-Value	LoA lower bound [95% CI]	LoA upper bound [95% CI]	MDC	SAD [95% CI]	SMAPE [95% CI]	ICC [95% CI]	Proportional bias	Heteroscedasticity	Log-transform applicable
Total sleep time (TST, minutes)	AWS-A	46.95 (67.26) [40.15, 53.74]	<.001	−84.88 [−96.50, −73.26]	178.78 [167.16, 190.4]	131.83	0.57 [0.47, 0.67]	7.30 [6.45, 8.16]	0.61 [0.51, 0.71]	True	True	False
	WSA-A	92.71 (81.16) [83.58, 101.84]	<.001	−66.37 [−81.99, −50.75]	251.79 [236.17, 267.41]	159.08	1.21 [1.09, 1.32]	14.31 [13.06, 15.57]	0.20 [0.15, 0.26]	False	False	False
	Emfit-A	101.47 (75.95) [91.11, 111.83]	<.001	−47.40 [−65.13, −29.67]	250.33 [232.61, 268.06]	148.87	1.28 [1.14, 1.41]	13.60 [12.26, 14.94]	0.22 [0.13, 0.31]	True	True	False
Sleep onset latency (SOL, minutes)	AWS-A	3.19 (32.95) [−0.14, 6.52]	.06	−61.39 [−67.08, −55.69]	67.77 [62.07, 73.46]	64.58	0.66 [0.56, 0.77]	57.25 [53.26, 61.23]	0.20 [0.13, 0.28]	False	False	False
	WSA-A	13.27 (30.30) [9.86, 16.68]	<.001	−46.13 [−51.96, −40.29]	72.66 [66.83, 78.49]	59.39	1.05 [0.93, 1.16]	61.14 [57.32, 64.97]	0.08 [0.02, 0.13]	False	True	False
	Emfit-A	8.74 (27.12) [5.04, 12.44]	<.001	−44.42 [−50.75, −38.09]	61.90 [55.57, 68.23]	53.16	1.16 [1.02, 1.30]	63.96 [59.48, 68.43]	0 [−0.04, 0.03]	True	True	False
Wake after sleep onset (WASO, minutes)	AWS-A	23.99 (68.19) [17.11, 30.88]	<.001	−109.66 [−121.44, −97.88]	157.64 [145.86, 169.43]	133.65	0.55 [0.45, 0.65]	25.11 [22.23, 27.99]	0.52 [0.43, 0.60]	True	True	False
	WSA-A	−44.70 (65.31) [−52.05, −37.35]	<.001	−172.71 [−185.28, −160.14]	83.32 [70.75, 95.89]	128.01	1.16 [1.05, 1.27]	57.31 [53.84, 60.78]	0.06 [0, 0.12]	—	—	True
	Emfit-A	−4.70 (54.43) [−12.12, 2.73]	.21	−111.38 [−124.09, −98.68]	101.99 [89.29, 114.70]	106.69	0.87 [0.74, 1.01]	32.67 [29.33, 36.00]	0.02 [−0.05, 0.09]	—	—	True
Sleep efficiency (SEFF, %)	AWS-A	−2.38 (10.06) [−3.39, −1.36]	<.001	−22.09 [−23.82, −20.35]	17.33 [15.59, 19.07]	19.71	0.47 [0.37, 0.57]	4.69 [4.13, 5.25]	0.59 [0.50, 0.67]	False	False	False
	WSA-A	9.19 (14.26) [7.59, 10.8]	<.001	−18.76 [−21.51, −16.02]	37.15 [34.4, 39.89]	27.96	1.02 [0.91, 1.14]	8.78 [7.78, 9.77]	0.12 [0.07, 0.17]	False	False	False
	Emfit-A	9.41 (11.05) [7.91, 10.92]	<.001	−12.25 [−14.83, −9.67]	31.07 [28.49, 33.65]	21.66	1.17 [1.04, 1.31]	6.74 [5.78, 7.70]	0.11 [0.04, 0.18]	True	True	True

All device measures were automatically generated by the device software [AP-A]. The values shown are the mean followed by the (standard deviation) and [95% confidence interval]. The metrics include Bias—difference in measurement between the Device and AWS|SD (Device – AWS|SD); *p*-value indicating the significant difference in the metric estimation tested using paired *t*-test; lower and upper bounds of the Bias; minimum detectable change (MDC)—smallest detectable change independent of measurement error (half of Bland–Altman agreement width); standardized absolute difference (SAD)—directionless version of Cohen’s *d*; symmetric mean absolute percentage error (SMAPE)—mean error in measurement expressed as percentage; absolute intraclass correlation with two-way random effects (ICC)—measures of measurement reliability; presence of proportional bias and deviation from homoscedasticity (heteroscedasticity); indication of log transform applicable to the data. The ICC estimates were computed for each participant and the mean, and 95% confidence interval is reported. The number of participants (days) used in each of the devices: AWS—34 (379), WSA—27 (306), and Emfit—16 (205). All the values are rounded to two decimal places.

When not using sleep diary information (AP-A), both CSTs overestimated TST (Bias: >90 minutes), SOL (Bias: >8 minutes), and SEFF (Bias: ≈9 %), while WASO was underestimated by WSA (Bias: −44 minutes), relative to AWS|SD. When not using the sleep diary, the AWS (AWS-A) overestimated TST (Bias: ≈47 minutes) and WASO (Bias: 24 minutes) while underestimating SEFF (Bias: ≈ −2%). The SOL estimates of AWS-A and WASO estimates of Emfit were in close agreement with the AWS|SD estimates with no significant differences. Ranking of the devices using SAD and SMAPE estimated against AWS|SD revealed that, for these nocturnal sleep measures, the AWS-A performed better compared to the CSTs (see Supplementary Figure 5). Among the CSTs, WSA was better than Emfit in estimating SOL while Emfit was better at WASO. The TST and SEFF estimation concordances of both CSTs were identical.

When sleep diary information was used to define the analysis period (AP-SD) for the CSTs, their performance improved (see Figure 3, Supplementary Figure 4, and Supplementary Tables 2 and 3). Across all-night sleep measures, the Emfit had better agreement with AWS|SD than the WSA.

### Epoch-by-epoch concordance of nocturnal in-bed periods

**Figure 4.**
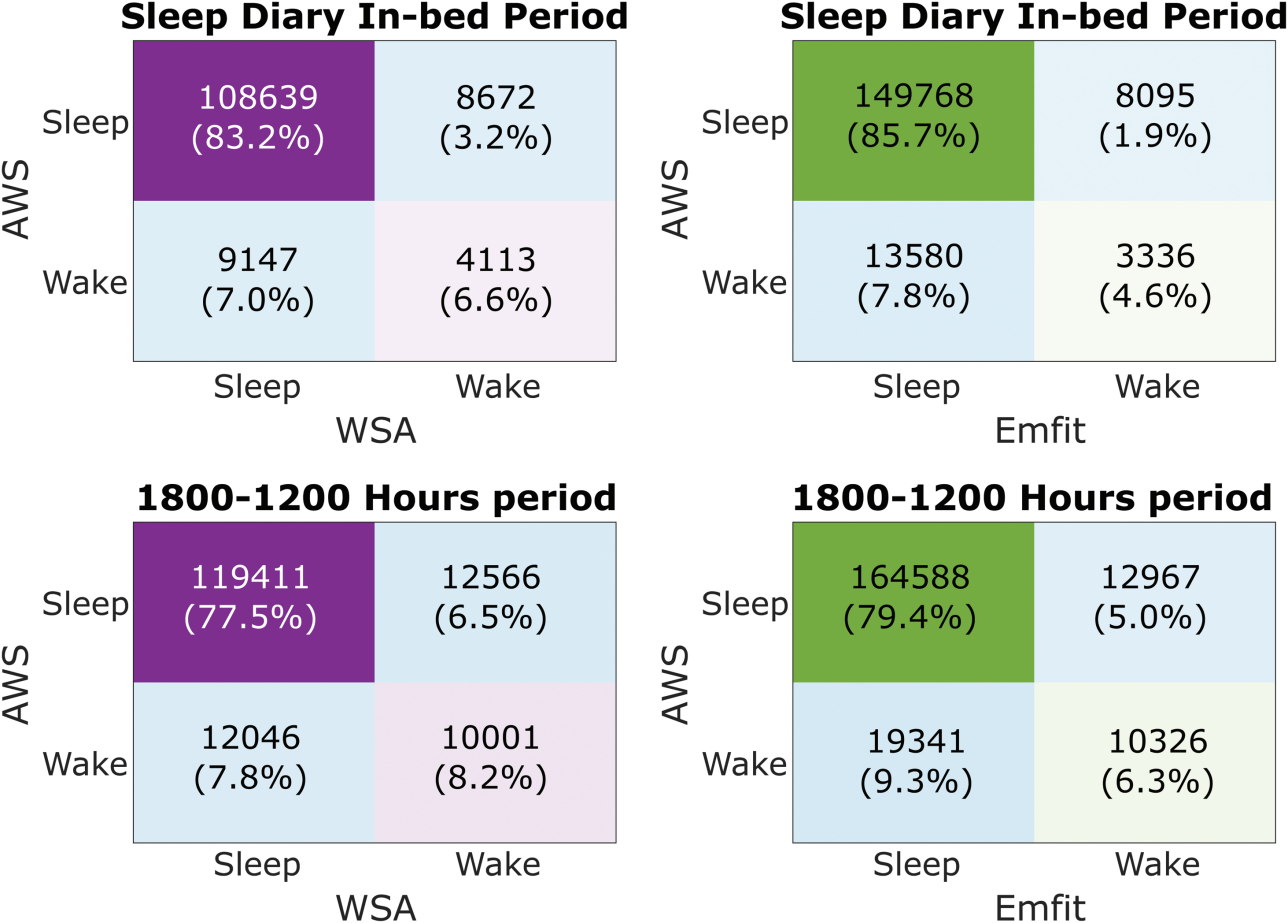
Pooled confusion matrices. The pooled confusion matrices are derived by summing participant wise EBE concordance confusion matrices. The panels on the top indicate the matrices computed over the sleep diary lights-off period (AP-SD) and the panel on the bottom indicate the period between 18:00 and 12:00 (hh:mm). The percentage in the confusion matrices depicts the percentages of true and false positive and negatives with respect to the total data. Total number of epochs for each device for the AP-SD (WSA—30571; Emfit—174779) and period between 18:00 and 12:00 hours (WSA—154024; Emfit—207222). The number of participants used in each of the devices is WSA [*n* = 27] and Emfit [*n* = 16].

The EBE concordance for sleep versus wake classification of the CSTs with AWS was determined over two different intervals: sleep diary in-bed period and the period between 18:00 and 12:00 hours (EBE concordance: Table 5 and pooled confusion matrices: Figure 4). For both analysis intervals, both the WSA and Emfit provided high sensitivity (>0.9) and low specificity (<0.4) with an accuracy greater than 0.8 and an MCC of <0.32.

**Table 5.** Epoch-by-epoch concordance

Sleep stage		Sensitivity	Specificity	Accuracy	Matthew’s CC	F1 Score
Sleep diary—in-bed period (AP-SD)						
Sleep/wake	WSA	0.92(0.10) [0.91, 0.93]	0.26(0.20) [0.24, 0.29]	0.86(0.09) [0.85, 0.87]	0.21(0.15) [0.2, 0.23]	0.92(0.07) [0.91, 0.93]
	Emfit	0.95(0.04) [0.94, 0.95]	0.21(0.15) [0.19, 0.23]	0.88(0.05) [0.87, 0.88]	0.18(0.11) [0.17, 0.20]	0.93(0.03) [0.93, 0.94]
Period between 18:00 and 12:00 hours						
Sleep/wake	WSA	0.90(0.10) [0.89, 0.91]	0.39(0.22) [0.36, 0.41]	0.84(0.09) [0.83, 0.85]	0.30(0.19) [0.28, 0.33]	0.90(0.07) [0.89, 0.91]
	Emfit	0.93(0.03) [0.92, 0.93]	0.38(0.14) [0.36, 0.40]	0.85(0.06) [0.84, 0.85]	0.31(0.14) [0.29, 0.33]	0.91(0.04) [0.90, 0.92]

The values shown are the mean followed by the (standard deviation) and [95% confidence interval].

### Daytime naps

Both the automated device estimates (AWS-A, WSA-A, and Emfit-A) of daytime sleep periods and the naps estimated using the EBE bed presence information from the WSA (WSA-BP) were compared to the sleep-diary-reported naps (ground truth). In cohort 1 (*n* = 18), sleep diary information on the occurrence and duration of nap was collected. In cohort 2 (*n* = 17), the sleep diary also contained information on the timing and location of the naps. Out of 35 participants (cohort 1 + cohort 2), 12 participants did not report any naps. On average, the participants (*n* = 23) who reported daytime naps took ≈1 nap per day (0.99 ± 0.31 naps/day (mean ± *SD*); range: [0.33, 1.80]) for a duration of ≈45 minutes (45.74 ± 34.49 minutes; range: [10, 180]; *N* = 110).

We first explored the performance of the devices in the 2-hour exclusion periods before and after the nocturnal sleep period. According to the sleep diary, no naps were taken in these periods (data on timing only available for cohort 2). The AWS-A detected a much large number of naps in the 2-hour transition window compared to the CSTs. In fact, WSA-A did not report any naps that generated a summary in this region although in-bed periods were detected (cohort 1: AWS-A = 86; WSA-A = 0; WSA-BP = 8 and cohort 2: AWS-A = 110; WSA-A = 0; WSA-BP = 33, and Emfit-A = 21).

The number of naps detected by AWS-A, after the exclusion of naps in the 2-hour transition windows before and after nocturnal sleep, was much larger than the number of naps reported in the sleep diary while the CSTs detected a smaller number of naps (cohort 1: Sleep diary = 57; AWS-A = 114; WSA-A = 5; WSA-BP = 15 and cohort 2: Sleep diary = 53; AWS-A = 98; WSA-A = 19; WSA-BP = 38 and Emfit-A = 30 [see Figure 6, A]).

Across cohorts 1 and 2, average nap duration estimated by AWS-A, WSA-A, WSA-BP, and Emfit-A were 61.29 ± 45.71 minutes (*N* = 208), 67.62 ± 38.90 minutes (*N* = 24), 53.09 ± 43.83 minutes (*N* = 53), and 62.74 ± 51.53 minutes (*N* = 30), respectively. In cohort 2, nap location was collected, and 35 naps occurred in bed and 19 naps were taken outside the bedroom. According to the sleep diary, the mean duration of the naps in bed was shorter 49.51 ± 34.21 minutes (range: [10, 180]) compared to naps not in bed, 63.68 ± 40.27 minutes (range: [10, 130]).

**Figure 5.**
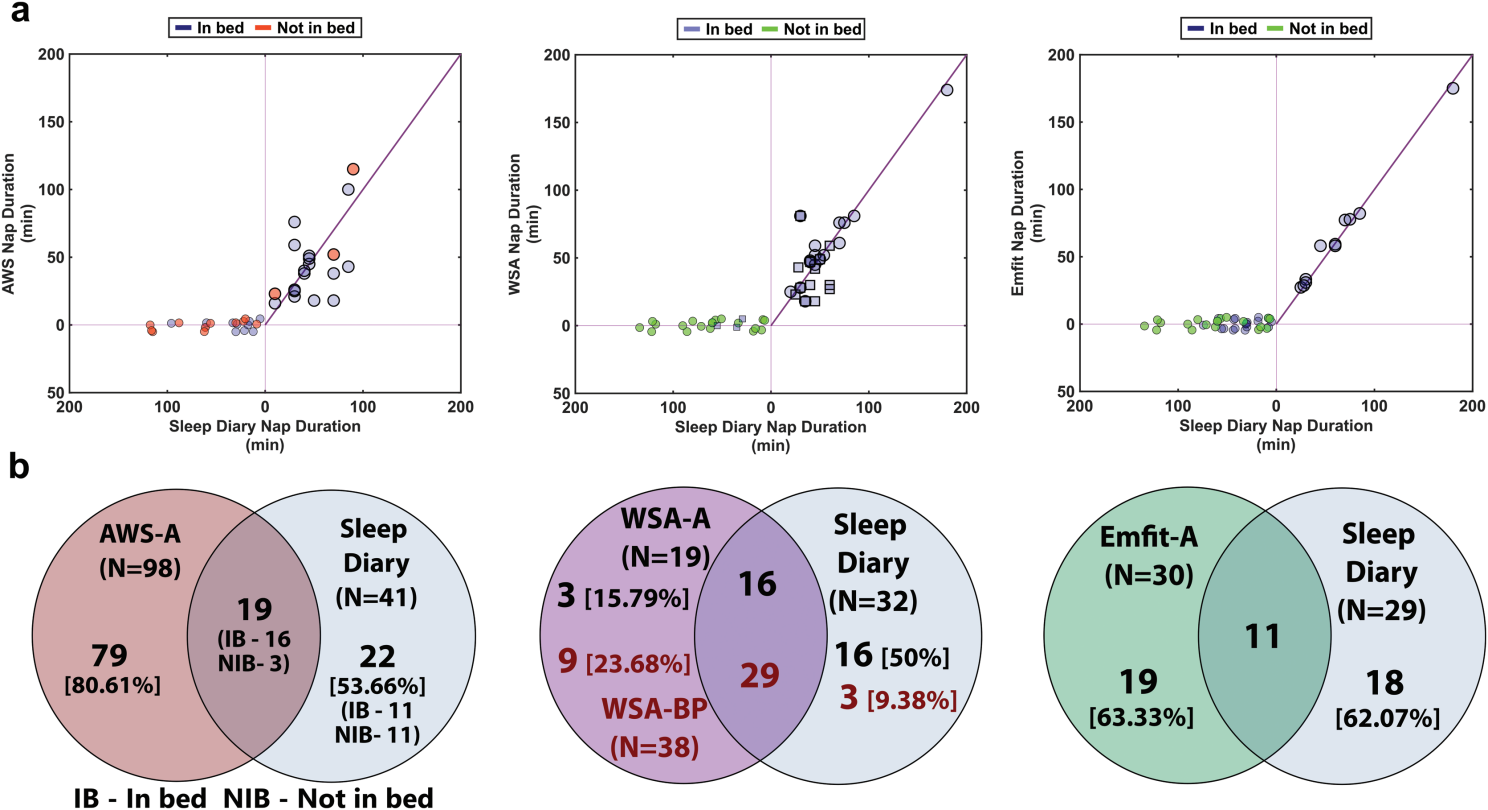
Nap estimation agreement between the device and the ground truth sleep diary. (A) The plots on the top depict the nap durations commonly available between the compared device and the sleep diary (XY quadrant). The data points depicted on the horizontal axis to the left of zero indicate the sleep diary nap events that were missed by the device. For WSA, naps detected automatically are indicated by circles while the naps detected via bed presence are indicated by squares. (B) The Venn diagrams on the bottom depict the portion of naps detected by the different devices compared to sleep diary. The overlapping regions are the accurately detected naps and the nonoverlapping naps regions on side of the device indicates unreported naps and those on the side of the sleep diary indicate naps missed by the device.

Since, in cohort 2, we also collected information on the timing of the naps we could estimate whether the naps detected by the devices were concordant with the sleep diary. Of the naps reported in the sleep diary to have occurred in bed, the AWS-A detected 46.34%, WSA-A detected 31.37%, WSA-BP detected 90.62%, and Emfit-A detected 22.92% of naps, respectively (Figure 5). In Figure 5, for WSA, the naps in bed detected automatically (WSA-A) are denoted by circles while the additional naps detected via bed presence (WSA-BP) are denoted by squares. The detailed summary of the distribution of the naps and the agreement of the duration estimates are depicted in Tables 6 and 7. Nap duration agreement of the CSTs with the sleep diary was higher (ICC: >0.85) than agreement of the AWS-A (ICC: 0.35) with the sleep diary. Among the CSTs, WSA-BP had the highest agreement (Bias:1.17 minutes) and missed a small portion of naps (*N* = 3) followed by Emfit-A (Bias:1.80 minutes) and WSA-A (Bias:3.63 minutes).

**Table 6.** Incidence of naps events in cohort 2

Device	Sleep diary naps			Device naps				
	Total naps, ie, ground truth [K] (Count)	Total in bed	Total not in bed	Total naps recorded [T] (Count)	Detected			Unreported naps recorded only in the device (Count (%[T]))
					Naps in bed (Count [% total in bed])	Naps not in bed (Count [%Total not in bed])	Total (Count [% {K}])	
AWS-A	41	27	14	98	16 (59.26)	3 (21.43)	19 (46.34)	79 (80.61)
WSA-A	51	32	19	19	16 (50)	0 (0)	16 (31.37)	3 (15.79)
WSA-BP	51	32	19	38	29 (90.62)	0 (0)	29 (56.86)	9 (23.68)
Emfit-A	48	29	19	30	11 (37.93)	0 (0)	11 (22.92)	19 (63.33)

The total number of participants in cohort 2 were 17; T—total numbers of naps recorded by the device; K—total number of naps in sleep diary that fall in the commonly available recording days. All naps detected in the 2-hour period prior to the time of *going to bed* time and after the time of *getting out of bed* time were removed to avoid the effect of sleep transition period. Following this, nap less than 10 minutess and over 300 minutes were also filtered from the analysis. WSA-BP represents the daytime bed presence-based nap analysis performed on the WSA bed presence information. All the values are rounded to two decimal places.

**Table 7.** Nap duration agreement metrics

In-bed nap duration	Device, Mean (*SD*)	Sleep diary, Mean (*SD*)	Bias (*SD*), [95% CI]	*p*-Value	LoA lower bound [95% CI]	LoA upper bound [95% CI]	MDC	SAD [95% CI]	SMAPE [95% CI]	ICC [95% CI]
AWS-A	41.44 (22.79)	45.94 (21.39)	−4.50 (25.28) [−17.97, 8.97]	.49	−54.05 [−77.58, −30.53]	45.05 [21.53, 68.58]	49.55	0.83 [0.31, 1.35]	20.17 [10.24, 30.09]	0.35 [−0.16, 0.83]
WSA-A	60.75 (35.76)	57.13 (37.38)	3.63 (14.70) [−4.21, 11.46]	.34	−25.19 [−38.87, −11.51]	32.44 [18.76, 46.12]	28.81	0.25 [−0.27, 0.76]	9.31 [2.65, 15.98]	0.92 [0.79, 0.99]
WSA-BP	53.65 (32.70)	52.48 (32.91)	1.17 (16.21) [−5.84, 8.18]	.73	−30.6 [−42.76, −18.44]	32.95 [20.79, 45.10]	31.77	0.31 [−0.12, 0.73]	11.03 [5.22, 16.84]	0.88 [0.74, 0.97]
Emfit-A	64.35 (42.14)	62.55 (44.22)	1.80 (5.02) [−1.57, 5.17]	.26	−8.03 [−13.98, −2.08]	11.63 [5.69, 17.58]	9.83	0.09 [−0.55, 0.73]	3.35 [0.98, 5.71]	0.99 [0.98, 1.0]

WSA-BP represents the daytime bed presence-based nap analysis performed on the WSA bed presence information. The values shown are the mean followed by the (standard deviation) and [95% confidence interval]. The metrics include Bias – difference in measurement between the Device and Sleep diary (Device—Sleep diary); *p*-value indicating the significant difference in the metric estimation tested using paired *t*-test; lower and upper bounds of the Bias; minimum detectable change (MDC)—smallest detectable change independent of measurement error (half of Bland–Altman agreement width); standardized absolute difference (SAD)—directionless version of Cohen’s *d*; symmetric mean absolute percentage error (SMAPE)—mean error in measurement; absolute intraclass correlation with two-way random effects (ICC)—measures of measurement reliability. The number of data points in each of the device is AWS-A—16; WSA-A—16; WSA-BP—29; Emfit-A—11. All the values are rounded to two decimal places.

#### Nap duration as percentage of 24-hour TST.

We computed the nap duration estimated by the different approaches (AWS-A, WSA-A, WSA-BP, and Emfit-A) as a percentage of 24-hour TST to determine the contribution of daytime naps to the 24-hour TST estimate. According to the sleep diary, naps contributed 12.97% ± 9.13% (mean ± *SD*) to the 24-hour TST. Among the compared devices, the WSA-A estimates of the contribution of naps to 24-hour TST (12.84% ± 6.11%) were close to the sleep diary estimate, whereas WSA-BP (10.91 ± 7.55%) underestimated this contribution and AWS-A (15.61% ± 9.87%) and Emfit-A (17.16% ± 11.11%) overestimated it. Please note that only the automatic all-night TST estimate was used for all the devices.

### 24-Hour sleep summary

**Figure 6.**
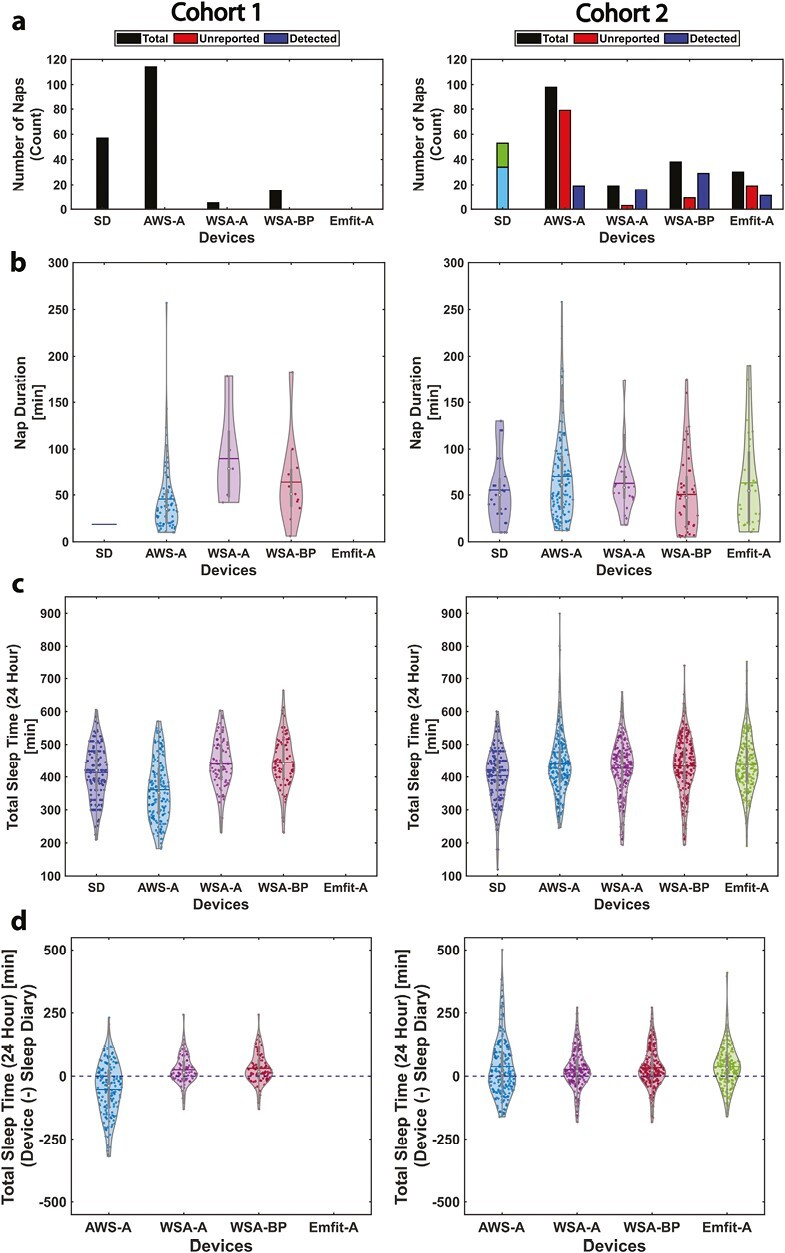
The 24-hour estimates of sleep and naps. (A) Number of naps recorded, unreported naps, and naps accurately detected by the device. For cohort 2, sleep diary (SD): blue—naps in bed and green—naps not in bed; WSA-A naps automatically recorded; WSA-BP naps detected using bed presence. (B, C) Nap duration (B) followed by TST (C). The blue horizontal line depicts the mean value, and(D) depicts the differences in the 24-hour TST estimates obtained from the devices compared to the sleep diary information.

The nap counts, nap durations, TST (all night), and 24-hour TST estimated for cohorts 1 and 2 are depicted in Figure 6. For WSA, both automatic naps and naps detected using bed presence information were used for 24-hour TST estimation separately. The sleep diary information on both daytime nap timing and duration was available only in cohort 2 and hence the data from cohort 1 (left panels) and cohort 2 (right panels) are plotted separately in Figure 6. In cohort 1, the Actiwatch (AWS-A) underestimated the 24-hour TST while WSA-A and WSA-BP overestimated it (Table 8). All the compared devices overestimated the 24-hour TST (Bias: >22 minutes) compared to sleep diary. The error in the 24-hour sleep duration estimate measure using SMAPE was about 10% for all devices (AWS-A,WSA-A, WSA-BP, and Emfit-A) compared to sleep diary. When compared across all used agreement measures, WSA-A performed better than the compared devices in both cohorts 1 and 2. In cohort 2, the performance of WSA-A was followed by WSA-BP, Emfit-A, and AWS-A.

**Table 8.** The 24-hour total sleep time agreement metrics

Total sleep time (24-hour estimate)		Device, Mean (*SD*)	Sleep diary, Mean (*SD*)	Bias (*SD*), [95% CI]	*p*-Value	LoA lower bound [95% CI]	LoA upper bound [95% CI]	MDC	SAD [95% CI]	SMAPE [95% CI]	ICC [95% CI]
Cohort 1 (*N* = 18)	AWS-A	360.31 (92.34)	413.29 (81.72)	−57.4 (108.09) [−74.39, −40.42]	<.001	−269.27 [−298.35, −240.18]	154.46 [125.37, 183.55]	211.86	1.08 [0.93, 1.24]	12.87 [11.25, 14.5]	0.26 [0.19, 0.34]
	WSA-A	437.85 (76.17)	412.36 (83.53)	25.48 (57.86) [13.00, 37.96]	<.001	−87.92 [−109.33, −66.51]	138.89 [117.48, 160.3]	113.41	0.56 [0.35, 0.78]	5.70 [4.43, 6.97]	0.58 [0.47, 0.68]
	WSA-BP	443.19 (79.91)	412.36 (83.53)	30.83 (61.89) [17.48, 44.17]	<.001	−90.47 [−113.38, −67.57]	152.13 [129.22, 175.03]	121.3	0.62 [0.40, 0.83]	6.26 [4.93, 7.58]	0.54 [0.44, 0.63]
Cohort 2 (*N* = 17)	AWS-A	441.96 (99.06)	403.87 (83.04)	38.09 (118.58) [21.84, 54.34]	<.001	−194.33 [−222.15, −166.52]	270.51 [242.69, 298.32]	232.42	0.99 [0.86, 1.13]	10.86 [9.45, 12.27]	0.18 [0.08, 0.27]
	WSA-A	429.97 (87.49)	403.87 (83.04)	26.05 (79.63) [15.11, 36.99]	<.001	−130.02 [−148.74, −111.29]	182.11 [163.39, 200.84]	156.07	0.72 [0.58, 0.86]	8.13 [6.95, 9.32]	0.34 [0.25, 0.42]
	WSA-BP	436.86 (91.21)	403.87 (83.04)	33.15 (80.9) [22.03, 44.26]	<.001	−125.41 [−144.44, −106.39]	191.71 [172.68, 210.73]	158.56	0.74 [0.60, 0.87]	8.36 [7.16 9.55]	0.32 [0.23, 0.40]
	Emfit-A	443.01 (85.52)	405.08 (79.62)	37.93 (85.46) [25.21, 50.64]	<.001	−129.58 [−151.35 −107.81]	205.43 [183.66, 227.20]	167.51	0.84 [0.70, 0.99]	8.36 [7.25, 9.48]	0.25 [0.14, 0.35]

*N* represents the number of participants in each cohort 1. WSA-BP represents the daytime bed presence-based nap analysis performed on the WSA bed presence information. The numbers below each device represents number of participants [total number of days]. The values shown are the mean followed by the (standard deviation) and [95% confidence interval]. The metrics include Bias—difference in measurement between the Device and Sleep diary (Device—Sleep diary); *p*-value indicating the significant difference in the metric estimation tested using paired *t-*test; lower and upper bounds of the Bias; minimum detectable change (MDC)—smallest detectable change independent of measurement error (half of Bland–Altman agreement width); standardized absolute difference (SAD)—directionless version of Cohen’s *d*; symmetric mean absolute percentage error (SMAPE)—mean error in measurement; absolute intraclass correlation with two-way random effects (ICC)—measures of measurement reliability. The ICC estimates were computed for each participant and the mean, and 95 % confidence interval is reported. All the values are rounded to two decimal places.

## Discussion

This study documents the strengths and limitations of CSTs in quantifying daytime and nocturnal sleep behavior longitudinally in older people at home, comparing them to a standard wearable actigraphy and a consensus sleep diary. The study data were collected from a diverse population of older adults with current and history of comorbidities. The participants had high level of functional ability and intact cognitive function. However, about half of the participants had severe or moderate sleep apnea, which is a widespread, under-reported health condition found commonly among older adults [30, 31]. These health conditions, commonly seen in an older adult population in the real world, contribute to the ecological validity of this study.

Previous longitudinal studies have employed CSTs for at-home monitoring of sleep behavior in older adult populations [18, 32–35] (Supplementary Caption 1). While these studies have demonstrated the value and scalability of CSTs for studying sleep behavior under various conditions such as COVID-19, PLWD, and health deteriorations, they did not use a standard sleep technology such as actigraphy combined with sleep diary information for evaluating the validity of the contactless sleep monitoring data. For instance, Kholghi et al. employed the Emfit QS to monitor sleep behavior in healthy older adults during COVID-19 while Eyal et al. demonstrated the use of Withings Sleep Analyser to identify abnormal nocturnal behavior in PLWD, but did not compare it to actigraphy.

Our analysis revealed that the CSTs accurately detected the time of *going to bed* and *getting out of bed*, and thus providing an accurate estimate of TIB duration during both the day and night, without the use of sleep diary information while actigraphy was less accurate. This is in line with the observations reported by Piantino et al. and van Rijssen et al. in younger populations, where bed sensors were compared to actigraphy and sleep diary [19, 36].

However, we found that the agreement between automatic estimates of CSTs and actigraphy-assisted sleep diary information for nocturnal sleep parameters such as sleep efficiency and SOL is modest. Our results indicate that when compared to AWS|SD, the WSA and Emfit provide similar nocturnal sleep assessment performance, as observed in previous studies involving similar devices [18, 19, 22, 36]. It is worth noting that WSA has not been previously evaluated under at-home conditions in older adults in any of the sleep assessment domains discussed, which makes it difficult to place our findings into the context of the existing literature. As for the Emfit device, a previous evaluation in older adults is available, but this was in a laboratory setting in which the device was compared to PSG and showed poor agreement [24]. Our evaluation of the WSA and Emfit against PSG in a laboratory study also demonstrated that both devices (WSA and Emfit) did not perform as well as diary-assisted actigraphy (AWS; Supplementary Caption 1).

At the epoch level, both WSA and Emfit were poor at discriminating between sleep and wake when compared to actigraphy. When the activity levels as measured by AWS were plotted for the sleep and wake states predicted by the CSTs, we noticed a clear difference in the activity levels between sleep and wake states while the distribution for the respective states remained close to identical between the CSTs (see Supplementary Figure 6). This can be seen as the device algorithm, at some level, primarily using activity to discriminate sleep and wake states and can be attributed to the poor performance of CSTs in existing literature [19, 24, 36].

Automatic detection of naps in a real-world setting is a major challenge and satisfactory solutions are essential for the quantitative description of 24-hour sleep–wake patterns. Our data, on the one hand, demonstrate that this still remains a challenge but, on the other hand, also shows that CSTs can provide reliable information on naps taken in bed and are less likely to overestimate the incidence of daytime napping compared to actigraphy without sleep diary information. Even though CSTs cannot detect naps taken out of bed, their TST estimate per 24 hours is more accurate compared to automatic estimates of actigraphy (AWS-A) if we accept the sleep diary data as ground truth. The higher number of naps detected by the AWS-A analysis observed in our study can be attributed to the lack of contextual information (eg, bed presence) in AWS and the use of inactivity by the AWS-automated algorithm to determine sleep periods. The WSA outperformed the compared devices and accurately determined bed presence during daytime naps taken in bed but did not generate automated summaries for nap periods determined to be wake bouts by the device algorithm. Overall, our data demonstrate the potential of CSTs for the at-scale longitudinal monitoring of sleep–wake cycles in older people.

### Limitations of this study

In our study, actigraphy combined with sleep diary information is used as a standard reference. Even though this approach is widely used in longitudinal sleep monitoring studies, actigraphy cannot be considered a gold-standard method for detecting sleep and wakefulness. This currently requires PSG. Another limitation of the study is the potential participant recall bias and the associated errors in the sleep diary information.

## Conclusions

The CSTs offer the ability to accurately detect 24-hour bed presence longitudinally and unobtrusively without the requirement for participants to provide additional information. The ability of the CSTs to accurately determine the sleep summary estimates such as TST, SOL, WASO, and SEFF is, however, unsatisfactory. Given the adherence issues related to wearable devices such as activity monitors, the unique characteristics of the CSTs make them, nevertheless, a reliable alternative to standard actigraphy devices for long-term monitoring of bed occupancy patterns particularly in community-dwelling older adult populations. Future efforts may be devoted to contactless detection of naps outside the bedroom and more accurate detection of wake within nocturnal TIB periods.

## Supplementary Material

zsad194_suppl_Supplementary_Materials

## Data Availability

The data used in this study are available from the coauthor Ciro della Monica upon reasonable request. Contact email: c.dellamonica@surrey.ac.uk.

## References

[1] Cross N , Terpening Z, Rogers NL, et al. Napping in older people “at risk” of dementia: relationships with depression, cognition, medical burden and sleep quality. J Sleep Res. 2015;24(5):494–502. doi:10.1111/jsr.12313 26096839

[2] Li P , Gao L, Yu L, et al. Daytime napping and Alzheimer’s dementia: a potential bidirectional relationship. Alzheimers Dementia. 2022;19:158–168. doi:10.1002/alz.12636 PMC9481741 35297533

[3] Owusu JT , Wennberg AMV, Holingue CB, Tzuang M, Abeson KD, Spira AP. Napping characteristics and cognitive performance in older adults. Int J Geriatr Psychiatry. 2019;34(1):87–96. doi:10.1002/gps.4991 30311961 PMC6445640

[4] Leng Y , Redline S, Stone KL, Ancoli-Israel S, Yaffe K. Objective napping, cognitive decline, and risk of cognitive impairment in older men. Alzheimers Dementia. 2019;15(8):1039–1047. doi:10.1016/j.jalz.2019.04.009 PMC6699896 31227429

[5] Winsky-Sommerer R , de Oliveira P, Loomis S, Wafford K, Dijk DJ, Gilmour G. Disturbances of sleep quality, timing and structure and their relationship with other neuropsychiatric symptoms in Alzheimer’s disease and schizophrenia: insights from studies in patient populations and animal models. Neurosci Biobehav Rev. 2019;97:112–137. doi:10.1016/j.neubiorev.2018.09.027 30312626

[6] Balouch S , Dijk DAD, Rusted J, Skene SS, Tabet N, Dijk D. Night-to-night variation in sleep associates with day-to-day variation in vigilance, cognition, memory, and behavioral problems in Alzheimer’s disease. Alzheimers Dementia. 2022;14(1):1–11. doi:10.1002/dad2.12303 PMC9109375 35603140

[7] Wang C , Holtzman DM. Bidirectional relationship between sleep and Alzheimer’s disease: role of amyloid, tau, and other factors. Neuropsychopharmacology. 2020;45(1):104–120. doi:10.1038/s41386-019-0478-5 31408876 PMC6879647

[8] Blackman J , Swirski M, Clynes J, Harding S, Leng Y, Coulthard E. Pharmacological and non-pharmacological interventions to enhance sleep in mild cognitive impairment and mild Alzheimer’s disease: a systematic review. J Sleep Res. 2021;30(4):1–20. doi:10.1111/jsr.13229 PMC8365694 33289311

[9] Livingston G , Huntley J, Sommerlad A, et al. Dementia prevention, intervention, and care: 2020 report of the Lancet Commission. Lancet. 2020;396(10248):413–446. doi:10.1016/S0140-6736(20)30367-6 32738937 PMC7392084

[10] Johnson DA , Jackson CL, Guo N, Sofer T, Laden F, Redline S. Perceived home sleep environment: associations of household-level factors and in-bed behaviors with actigraphy-based sleep duration and continuity in the Jackson Heart Sleep Study. Sleep. 2021;44(11). doi:10.1093/sleep/zsab163 PMC8678916 34283244

[11] Ancoli-Israel S , Cole R, Alessi C, Chambers M, Moorcroft W, Pollak CP. The role of actigraphy in the study of sleep and circadian rhythms. American Academy of Sleep Medicine Review Paper. Sleep. 2003;26(3):342–392. doi:10.1093/sleep/26.3.342 12749557

[12] Jones C , Moyle W. A feasibility study of DreampadTM on sleep, wandering and agitated behaviors in people living with dementia. Geriatr Nurs (Minneap). 2020;41(6):782–789. doi:10.1016/j.gerinurse.2020.04.014 32522427

[13] Moore K , O’Shea E, Kenny L, et al. Older adults’ experiences with using wearable devices: qualitative systematic review and meta-synthesis. JMIR Mhealth Uhealth. 2021;9(6):e23832. doi:10.2196/23832 34081020 PMC8212622

[14] Kennedy MR , Huxtable R, Birchley G, Ives J, Craddock I. “A question of trust” and “a leap of faith”-study participants’ perspectives on consent, privacy, and trust in smart home research: Qualitative study. JMIR Mhealth Uhealth. 2021;9(11):e25227. doi:10.2196/25227 34842551 PMC8665399

[15] Stenholm S , Kronholm E, Bandinelli S, Guralnik JM, Ferrucci L. Self-reported sleep duration and time in bed as predictors of physical function decline: results from the InCHIANTI study. Sleep. 2011;34(11):1583–1593. doi:10.5665/sleep.1402 22043129 PMC3198213

[16] Tsai LT , Boyle E, Brønd JC, et al. Associations between objectively measured physical activity, sedentary behaviour and time in bed among 75+ community-dwelling Danish older adults. BMC Geriatr. 2021;21(1):1–8. doi:10.1186/s12877-020-01856-6 33446107 PMC7807682

[17] Bankole A , Anderson MS, Homdee N, et al. BESI: behavioral and environmental sensing and intervention for dementia caregiver empowerment—Phases 1 and 2. Am J Alzheimers Dis Other Demen. 2020;35. doi:10.1177/1533317520906686 PMC10624017 32162529

[18] Kholghi M , Ellender CM, Zhang Q, Gao Y, Higgins L, Karunanithi M. Home-based sleep sensor measurements in an older australian population: Before and during a pandemic. Sensors. 2021;21(18):5993. doi:10.3390/s21185993. 34577202 PMC8471147

[19] Piantino J , Luther M, Reynolds C, Lim MM. Emfit bed sensor activity shows strong agreement with wrist actigraphy for the assessment of sleep in the home setting. Nat Sci Sleep. 2021;13:1157–1166. doi:10.2147/NSS.S306317 34295199 PMC8291858

[20] Chinoy ED , Cuellar JA, Huwa KE, et al. Performance of seven consumer sleep-tracking devices compared with polysomnography. Sleep. 2021;44(5). doi:10.1093/sleep/zsaa291 PMC8120339 33378539

[21] Siyahjani F , Molina GG, Barr S, Mushtaq F. Performance evaluation of a smart bed technology against polysomnography. Sensors. 2022;22(7):2605. doi:10.3390/s22072605 35408220 PMC9002520

[22] Stone JD , Rentz LE, Forsey J, et al. Evaluations of commercial sleep technologies for objective monitoring during routine sleeping conditions. Nat Sci Sleep. 2020;12:821–842. doi:10.2147/NSS.S270705 33149712 PMC7603649

[23] Lauteslager T , Kampakis S, Williams AJ, Maslik M, Siddiqui F. Performance evaluation of the Circadia contactless breathing monitor and sleep analysis algorithm for sleep stage classification. In: 2020 42nd Annual International Conference of the IEEE Engineering in Medicine & Biology Society (EMBC). IEEE; 2020: 5150–5153. doi:10.1109/EMBC44109.2020.9175419 33019145

[24] Kholghi M , Szollosi I, Hollamby M, Bradford D, Zhang Q. A validation study of a ballistocardiograph sleep tracker against polysomnography. J Clin Sleep Med. 2022;18(4):1203–1210. doi:10.5664/jcsm.9754 34705630 PMC8974370

[25] Carney CE , Buysse DJ, Ancoli-Israel S, et al. The consensus sleep diary: standardizing prospective sleep self-monitoring. Sleep. 2012;35(2):287–302. doi:10.5665/sleep.1642 22294820 PMC3250369

[26] Smith MT , McCrae CS, Cheung J, et al. Use of Actigraphy for the evaluation of sleep disorders and circadian rhythm sleep-wake disorders: an American academy of sleep medicine clinical practice guideline. J Clin Sleep Med. 2018;14(7):1231–1237. doi:10.5664/jcsm.7230 29991437 PMC6040807

[27] Menghini L , Cellini N, Goldstone A, Baker FC, de Zambotti M. A standardized framework for testing the performance of sleep-tracking technology: step-by-step guidelines and open-source code. Sleep. 2021;44(2). doi:10.1093/sleep/zsaa170 PMC7879416 32882005

[28] Euser AM , Dekker FW, le Cessie S. A practical approach to Bland-Altman plots and variation coefficients for log transformed variables. J Clin Epidemiol. 2008;61(10):978–982. doi:10.1016/j.jclinepi.2007.11.003 18468854

[29] Haghayegh S , Kang HA, Khoshnevis S, Smolensky MH, Diller KR. A comprehensive guideline for Bland–Altman and intra class correlation calculations to properly compare two methods of measurement and interpret findings. Physiol Meas. 2020;41(5):055012. doi:10.1088/1361-6579/ab86d6. 32252039

[30] Dunietz GL , Chervin RD, Burke JF, Conceicao AS, Braley TJ. Obstructive sleep apnea treatment and dementia risk in older adults. Sleep. 2021;44(9). doi:10.1093/sleep/zsab076 PMC8436135 33769542

[31] Gosselin N , Baril A-A, Osorio RS, Kaminska M, Carrier J. Obstructive sleep apnea and the risk of cognitive decline in older adults. Am J Respir Crit Care Med. 2019;199(2):142–148. doi:10.1164/rccm.201801-0204PP 30113864 PMC6943882

[32] Zhang G , Vahia IV, Liu Y, et al. Contactless in-home monitoring of the long-term respiratory and behavioral phenotypes in older adults with COVID-19: a case series. Front Psychiatry. 2021;12:12. doi:10.3389/fpsyt.2021.754169 PMC8580478 34777058

[33] Hashizaki M , Nakajima H, Shiga T, Tsutsumi M, Kume K. A longitudinal large-scale objective sleep data analysis revealed a seasonal sleep variation in the Japanese population. Chronobiol Int. 2018;35(7):933–945. doi:10.1080/07420528.2018.1443118 29589960

[34] Schütz N , Saner H, Botros A, et al. Contactless sleep monitoring for early detection of health deteriorations in community-dwelling older adults: exploratory study. JMIR MHealth UHealth. 2021;9(6):e24666. doi:10.2196/24666 34114966 PMC8235297

[35] Soreq E , Kolanko MA, Monica C, et al. Monitoring abnormal nocturnal behaviour in the homes of patients living with dementia. Alzheimers Dementia 2022;18(S2). doi:10.1002/alz.067936

[36] van Rijssen IM , Hulst RY, Gorter JW, et al. Device-based and subjective measurements of sleep in children with cerebral palsy: a comparison of sleep diary, actigraphy, and bed sensor data. J Clin Sleep Med. 2023;19(1):35–43. doi:10.5664/jcsm.10246. 35975545 PMC9806786

