# Supplementary material for: Contactless and longitudinal monitoring of nocturnal sleep and daytime naps in older men and women: a digital health technology evaluation study
Source: Sleep. 2023 Jul 20;46(10):zsad194. doi: 10.1093/sleep/zsad194 (PMC10566241; doi:10.1093/sleep/zsad194)
Supplement: zsad194_suppl_Supplementary_Materials [file zsad194_suppl_supplementary_materials.docx]

# Supplemental Materials

# Contactless and Longitudinal Monitoring of Nocturnal Sleep and Daytime Naps in Older Men and Women: A Digital Health Technology Evaluation Study

**Kiran KG Ravindran^1,2^, Ciro della Monica^1,2^, Giuseppe Atzori^1^, Damion Lambert^1,2^, Victoria Revell^1^ and Derk-Jan Dijk^1,2^**

1 Surrey Sleep Research Centre, University of Surrey, Guildford, United Kingdom.

2 UK Dementia Research Institute, Care Research and Technology Centre at Imperial College, London and the University of Surrey, Guildford, United Kingdom.

**Corresponding Author Details**

Name: Dr Kiran Kumar Guruswamy Ravindran

Address: Surrey Sleep Research Centre, University of Surrey, GU2 7XP

Email: k.guruswamyravindran@surrey.ac.uk

**Supplemental Caption 1:** Kiran K G Ravindran et al., Three Contactless Sleep Technologies Compared with Polysomnography and Actigraphy: An Observational Study in a Heterogenous Group of Older Men and Women in a Model of Mild Sleep Disturbance. JMIR Preprints. 07/02/2023:46338 doi: <http://doi.org/10.2196/preprints.46338>.


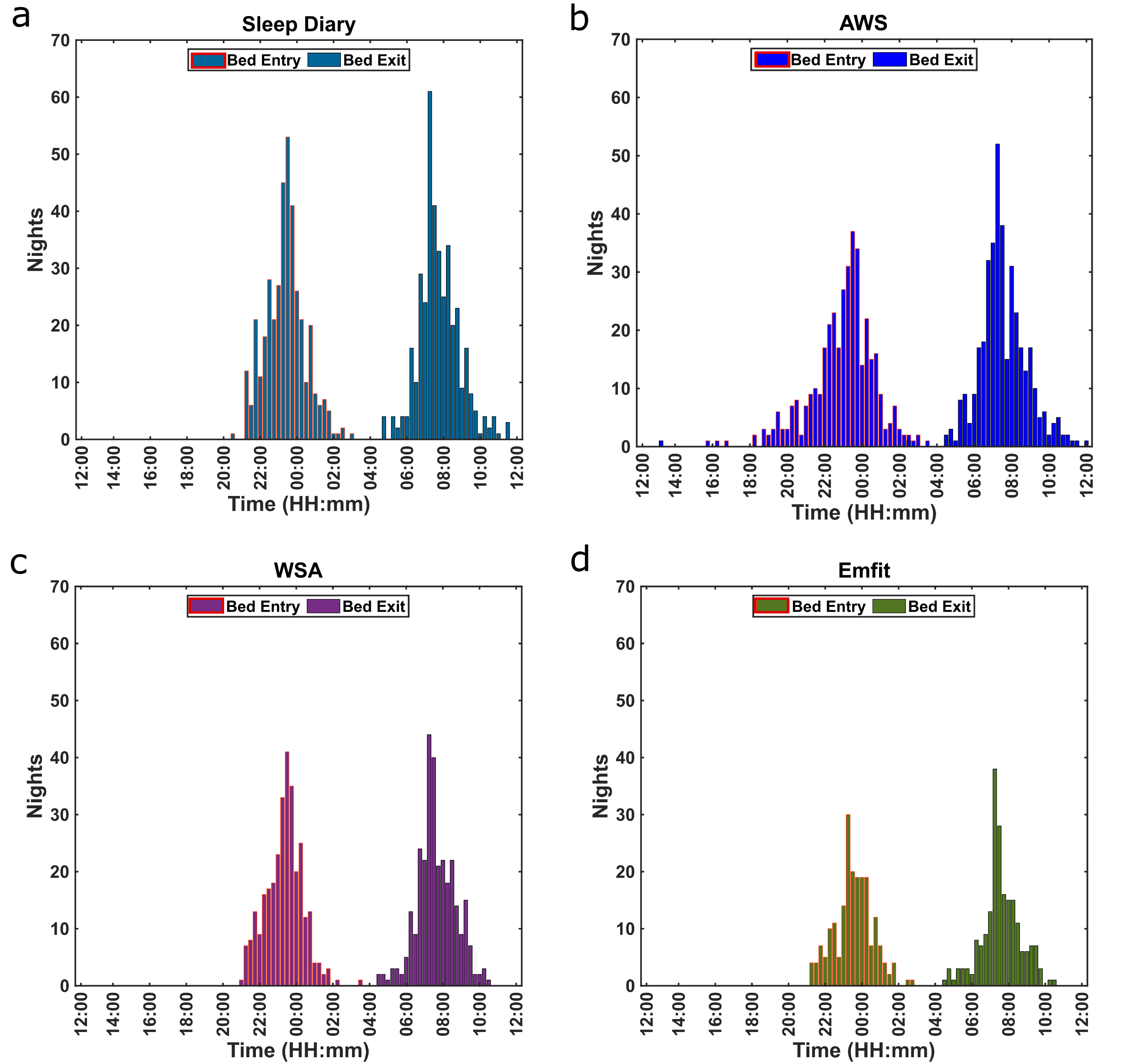


**Figure 1. Histograms representing the distribution of going to bed and getting out of bedtimes for the sleep diary (n=392), AWS-A (n=385), WSA (n=309) and Emfit (n=210).**


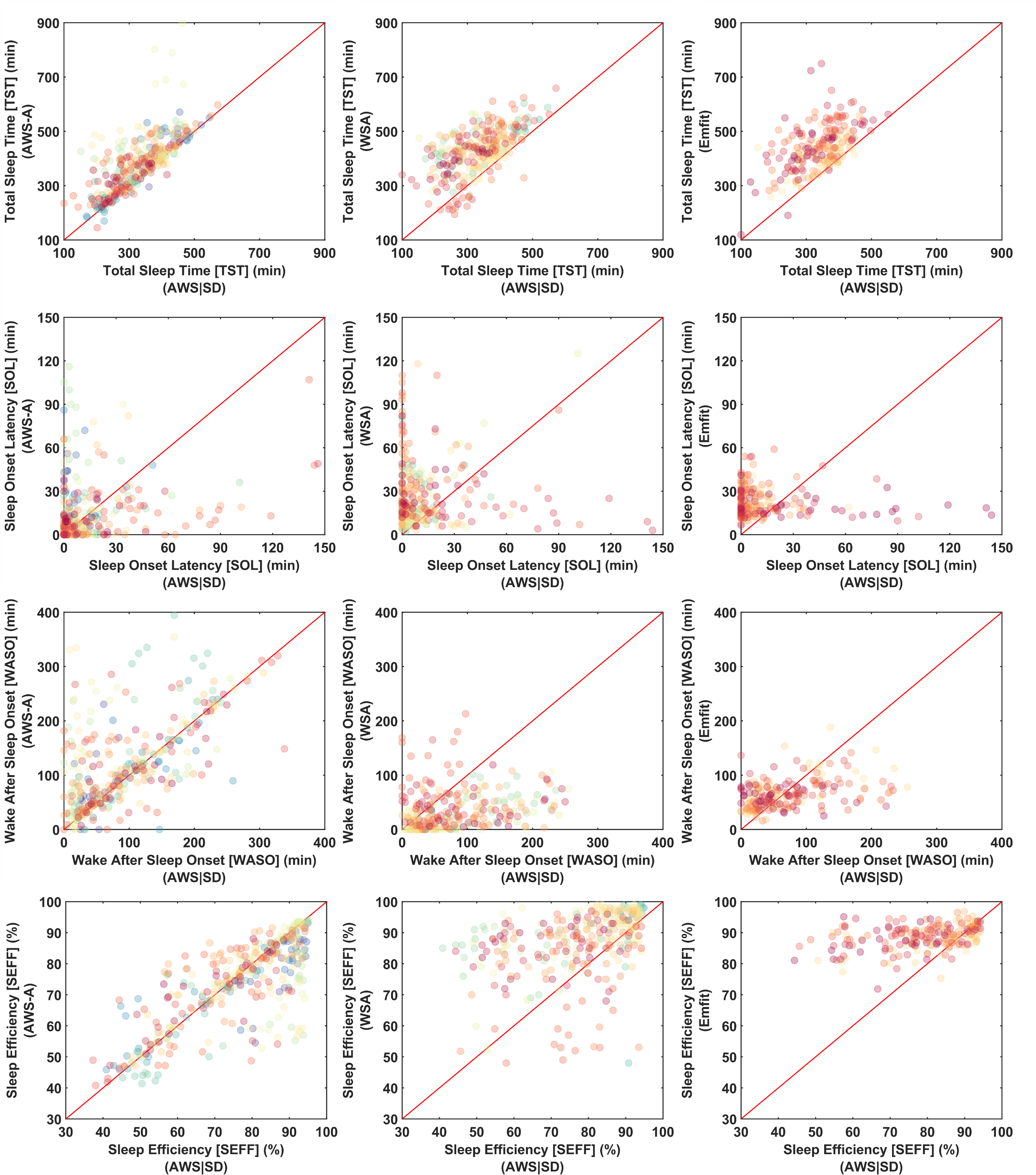


**Figure 2. Scatter plots of all-night sleep summary measure estimations.** The 45 degrees indicate the line of perfect agreement between the AWS|SD and device estimates. The data points are colour coded by each participant. All estimates are automatically generated by the respective devices without sleep diary information.


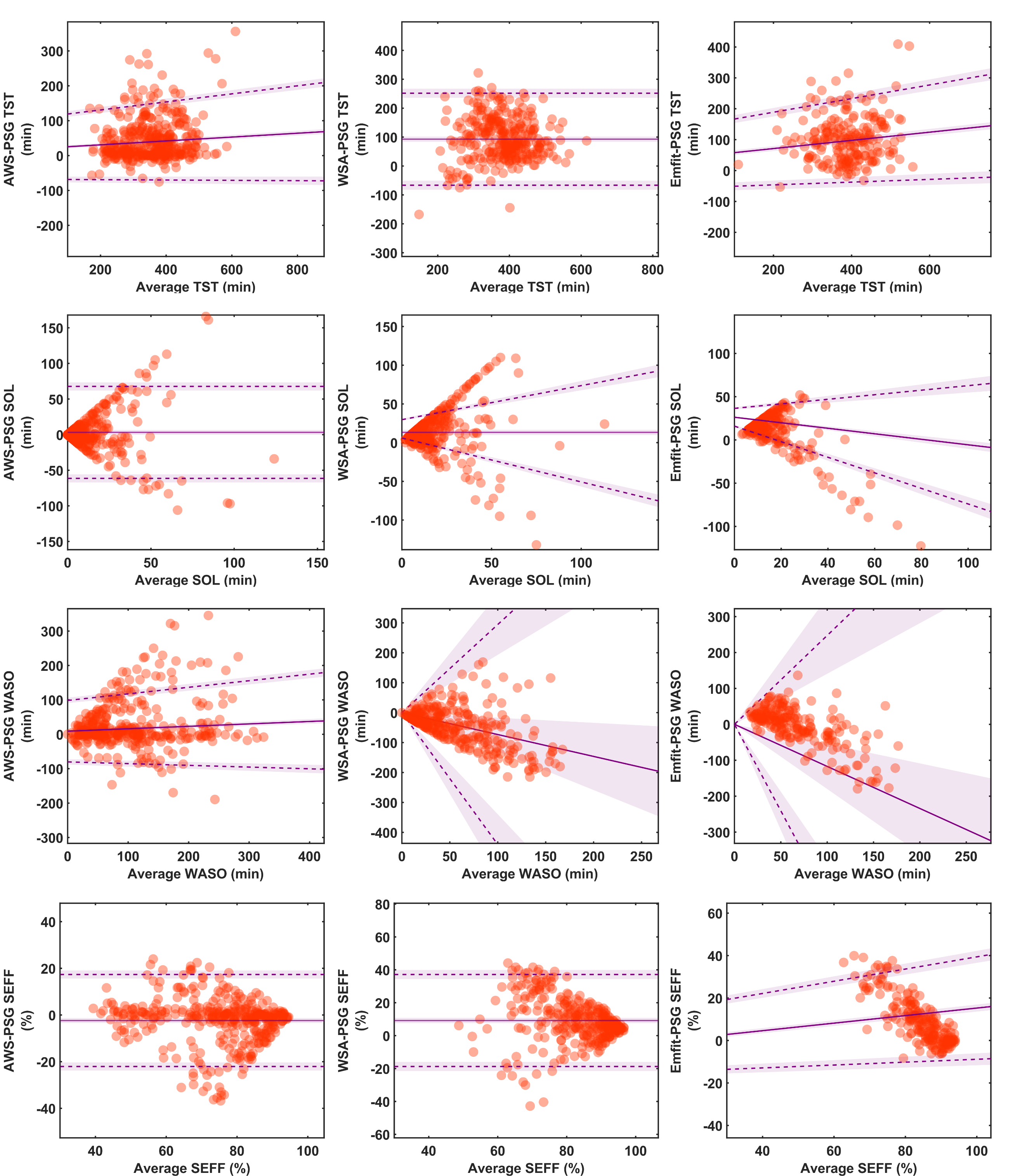


**Figure 3. Bland-Altman plots with corrections applied, for all-night sleep summary measure estimations.** All device measures are automatically generated by the device software. The type of correction applied on the device measure is provided in Table 1. The bias (solid line) and limits of agreement (dotted lines) are depicted with their 95% confidence intervals. The data points above the line indicate overestimations while points below the line indicate underestimations. The number of participants [days] used in each of the devices are AWS – 34 (392), WSA – 27 (306) and Emfit – 16 (205).

**Table 1. All-night sleep-wake summary measure agreement metrics (AP-A) – Corrected Bland- Altman measures.**

| **Sleep measure** | | **Proportional Bias** | **Heteroscedasticity** | **Log transform**  **Applicable** | **Device**  **Mean (SD)** | **AWS\|SD**  **Mean (SD)** | **Bias (SD)** | **Bias CI** | **LoA** | **LoA CI** |
| --- | --- | --- | --- | --- | --- | --- | --- | --- | --- | --- |
| **Total Sleep Time**  **(TST, min)** | **AWS** | True | True | False | 377.08 (97.73) | 330.13 (82.46) | 46.95+12.54 × Avg. | a0 =[18.15 52.55], a1=[6.92 41.34] | Bias ± 2.46 ( 46.81+5.43 × Avg.) | b0 =[9.36 50.73], b1=[1.50 42.88] |
|  | **WSA** | False | False | False | 426.57 (86.87) | 333.86 (83.14) | - | - | - | - |
|  | **Emfit** | True | True | False | 439.18 (84.38) | 337.71 (76.85) | 101.47+25.31× Avg. | a0 =[33.53 109.67], a1=[17.08 93.26] | Bias ± 2.46 (54.94+6.71 × Avg.) | b0 =[11.91 60.13], b1=[1.51 49.76] |
| **Sleep Onset Latency (SOL, min)** | **AWS** | False | False | False | 15.25 (28.39) | 12.06 (21.42) | - | - | - | - |
|  | **WSA** | False | True | False | 25.95 (21.90) | 12.69 (21.69) | 13.27 (30.30) | [9.86 16.68] | Bias ± 2.46 (19.09+8.33 × Avg.) | b0 =[10.40 21.15], b1=[6.27 17.02] |
|  | **Emfit** | True | True | False | 22.56 (9.75) | 13.82 (24.25) | 8.74+-10.17× Avg. | a0 =[-7.28 11.62], a1=[-13.05 5.86] | Bias ± 2.46 (16.82+7.40 × Avg.) | b0 =[9.36 18.78], b1=[5.43 14.86] |
| **Wake After Sleep Onset (WASO, min)** | **AWS** | True | True | False | 112.68 (81.76) | 88.69 (72.79) | 23.99+8.65× Avg. | a0 =[14.40 29.73], a1=[2.91 18.26] | Bias ± 2.46 (45.83+5.91 × Avg.) | b0 =[10.10 50.01], b1=[1.72 41.64] |
|  | **WSA** | - | - | True | 34.52 (36.21) | 79.22 (63.82) | -61.23+-77.95× Avg. | a0 =[-137.78 -108.23], a1=[-18.13 -14.24] | Bias ± 2.46 ( 408.64+248.60 × Avg.) | b0 =[261.71 421.72], b1=[235.49 395.55] |
|  | **Emfit** | - | - | True | 64.79 (27.00) | 69.49 (59.18) | -113.09+-122.64× Avg. | a0 =[-188.36 -173.69], a1=[-56.91 -52.48] | Bias ± 2.46 ( 423.64+276.51 × Avg.) | b0 =[289.77 436.86], b1=[263.26 410.42] |
| **Sleep Efficiency (SEFF, %)** | **AWS** | False | False | False | 73.42 (13.72) | 75.8 (15.05) | - | - | - | - |
|  | **WSA** | False | False | False | 86.43 (11.67) | 77.23 (13.77) | - | - | - | - |
|  | **Emfit** | True | True | True | 88.61 (4.02) | 79.19 (12.00) | 9.41+3.82× Avg. | a0 =[5.01 10.6], a1=[2.63 8.23] | Bias ± 2.46 ( 8.18+0.94 × Avg.) | b0 =[1.66 8.90], b1=[0.22 7.46] |


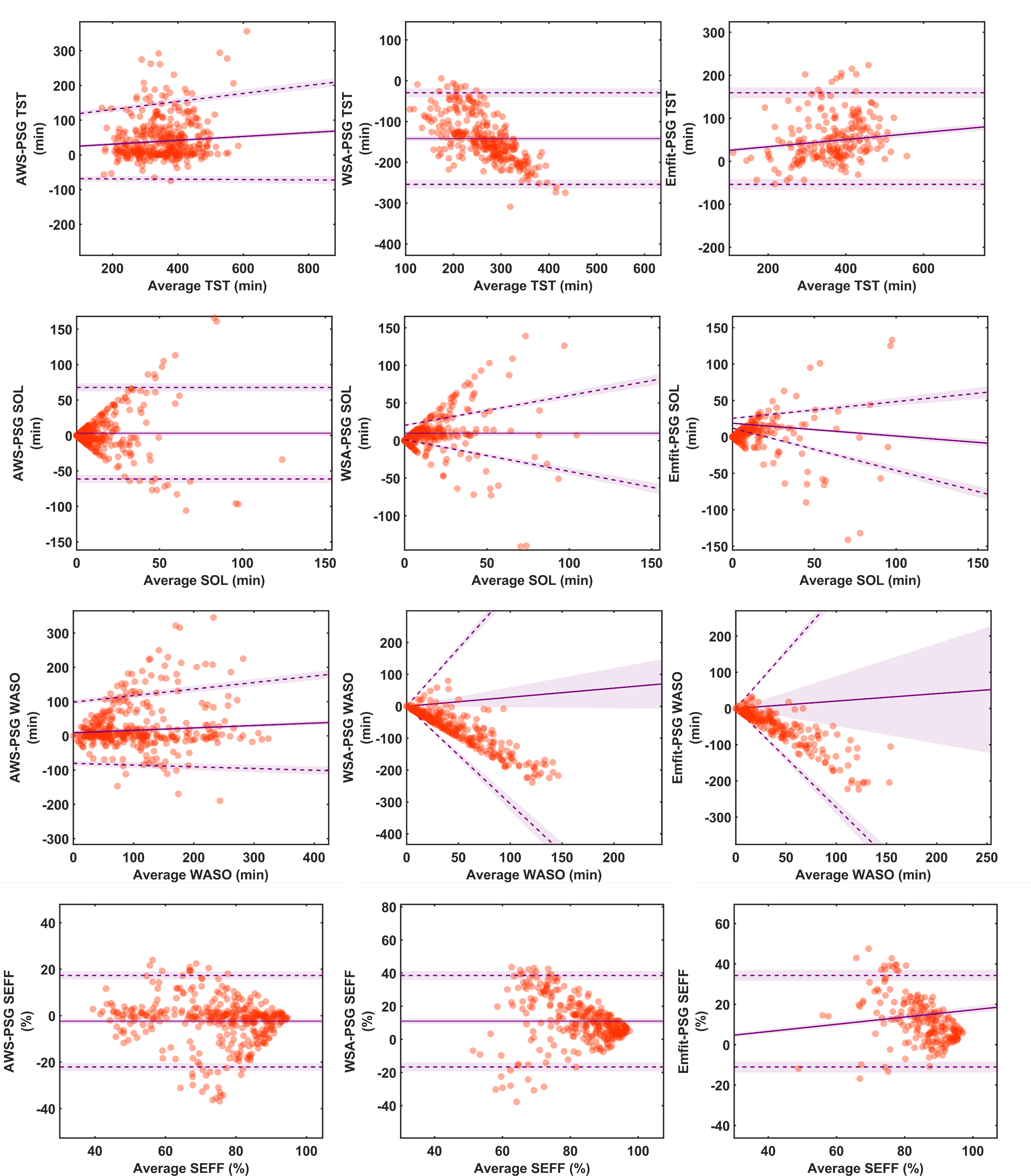


**Figure 4. All-night sleep summary measure estimations Bland-Altman plots (AP-SD) with corrections applied.** The AWS measures are automatically generated while the WSA and Emfit measures are generated using the timeseries data over the sleep diary lights off period. The type of correction applied on the device measure is provided in Table 2. The bias (solid line) and limits of agreement (dotted lines) are depicted with their 95% confidence intervals. The data points above the line indicate overestimations while points below the line indicate underestimations. The number of participants [days] used in each of the devices are AWS – 34 (392), WSA – 27 (306) and Emfit – 16 (205).

| **Sleep measure** | | **Bias**  **[95% CI]** | **p-value** | **LoA Lower bound**  **[95% CI]** | **LoA Upper bound**  **[95% CI]** | **MDC** | **SAD**  **[95% CI]** | **SMAPE**  **[95% CI]** | **ICC**  **[95% CI]** | **Proportional Bias** | **Heteroscedasticity** | **Log transform**  **Applicable** |
| --- | --- | --- | --- | --- | --- | --- | --- | --- | --- | --- | --- | --- |
| **Total Sleep Time**  **(TST, min)** | **AWS** | 46.95 (67.26)  [40.15 53.74] | <0.001 | -84.88  [-96.50 -73.26] | 178.78  [167.16 190.40] | 131.83 | 0.57  [0.47 0.67] | 7.3  [6.45 8.16] | 0.61  [0.51 0.71] | True | True | False |
|  | **WSA** | -141.76 (57.23)  [-148.20 -135.32] | <0.001 | -253.94  [-264.95 -242.92] | -29.58  [-40.60 -18.57] | 112.18 | 2.11  [2.00 2.22] | 26.78  [25.72 27.84] | 0.10  [0.08 0.12] | False | False | False |
|  | **Emfit** | 52.84 (54.31)  [45.43 60.24] | <0.001 | -53.60  [-66.28 -40.93] | 159.28  [146.60 171.96] | 106.44 | 0.68  [0.54 0.81] | 7.88  [6.94 8.83] | 0.50  [0.4 0.6] | True | False | False |
| **Sleep Onset Latency (SOL, min)** | **AWS** | 3.19 (32.95)  [-0.14 6.52] | 0.06 | -61.39  [-67.08 -55.69] | 67.77  [62.07 73.46] | 64.58 | 0.66  [0.56 0.77] | 57.25  [53.26 61.23] | 0.20  [0.13 0.28] | False | False | False |
|  | **WSA** | 9.75 (31.07)  [6.25 13.24] | <0.001 | -51.15  [-57.13 -45.17] | 70.65  [64.67 76.63] | 60.9 | 0.73  [0.62 0.84] | 55.73  [51.62 59.84] | 0.28  [0.19 0.37] | False | True | False |
|  | **Emfit** | 5.06 (32.06)  [0.69 9.43] | 0.023 | -57.77  [-65.25 -50.29] | 67.89  [60.41 75.38] | 62.83 | 0.62  [0.48 0.76] | 51.2  [46.05 56.34] | 0.22  [0.1 0.35] | True | True | False |
| **Wake After Sleep Onset (WASO, min)** | **AWS** | 23.99 (68.19)  [17.11 30.88] | <0.001 | -109.66  [-121.44 -97.88] | 157.64  [145.86 169.43] | 133.65 | 0.55  [0.45 0.65] | 25.11  [22.23 27.99] | 0.52  [0.43 0.6] | True | True | False |
|  | **WSA** | -67.03 (61.06)  [-73.9 -60.16] | <0.001 | -186.7  [-198.45 -174.95] | 52.65  [40.90 64.4] | 119.68 | 1.5  [1.39 1.61] | 77.53  [74.74 80.31] | 0.04  [0.02 0.06] | - | - | True |
|  | **Emfit** | -52.9 (53.79)  [-60.24 -45.57] | <0.001 | -158.32  [-170.88 -145.77] | 52.52  [39.97 65.08] | 105.42 | 1.27  [1.13 1.4] | 63.82  [59.99 67.65] | 0.07  [0.03 0.1] | - | - | True |
| **Sleep Efficiency (SEFF, %)** | **AWS** | -2.38 (10.06)  [-3.39 -1.36] | <0.001 | -22.09  [-23.82 -20.35] | 17.33  [15.59 19.07] | 19.71 | 0.47  [0.37 0.57] | 4.69  [4.13 5.25] | 0.59  [0.5 0.67] | False | False | False |
|  | **WSA** | 10.94 (14.11)  [9.35 12.53] | <0.001 | -16.71  [-19.42 -13.99] | 38.59  [35.87 41.3] | 27.65 | 1.12  [1.01 1.24] | 9.22  [8.31 10.14] | 0.12  [0.07 0.17] | False | False | False |
|  | **Emfit** | 11.63 (11.57)  [10.05 13.21] | <0.001 | -11.04  [-13.74 -8.34] | 34.30  [31.60 37.00] | 22.67 | 1.26  [1.12 1.4] | 7.99  [7.04 8.94] | 0.16  [0.08 0.23] | True | False | False |

**Table 2. All-night sleep-wake summary measure agreement metrics (AP-SD).**

**Table 3. All-night sleep-wake summary measure agreement metrics (AP-SD) - Corrected Bland- Altman measures.**

| **Sleep measure** | | **Proportional Bias** | **Heteroscedasticity** | **Log transform**  **Applicable** | **Device**  **Mean (SD)** | **AWS\|SD**  **Mean (SD)** | **Bias (SD)** | **Bias CI** | **LoA** | **LoA CI** |
| --- | --- | --- | --- | --- | --- | --- | --- | --- | --- | --- |
| **Total Sleep Time**  **(TST, min)** | **AWS** | True | True | False | 377.08 (97.73) | 330.13 (82.46) | 46.95+12.54× Avg. | a0 =[18.15 52.55], a1=[6.92 41.34] | Bias - 2.46 ( 46.81+5.43 × Avg.) | b0 =[9.36 50.73], b1=[1.50 42.88] |
|  | **WSA** | False | False | False | 192.10 (46.53) | 333.86 (83.14) | - | - | - | - |
|  | **Emfit** | True | False | False | 390.55 (91.07) | 337.71 (76.85) | 52.84+15.83× Avg. | a0 =[21.8 58.79], a1=[9.86 46.89] | Bias ±106.44 | Bias ±[93.77 119.11] |
| **Sleep Onset Latency (SOL, min)** | **AWS** | False | False | False | 15.25 (28.39) | 12.06 (21.42) | - | - | - | - |
|  | **WSA** | False | True | False | 22.43 (27.99) | 12.69 (21.69) | 9.75 (31.07) | [6.25 13.24] | Bias ± 2.46 ( 16.47+7.35 × Avg.) | b0 =[9.74 18.86], b1=[4.96 14.08] |
|  | **Emfit** | True | True | False | 18.88 (29.05) | 13.82 (24.25) | 5.06+-7.96× Avg. | a0 =[-4.39 8.62], a1=[-11.52 1.50] | Bias ± 2.46 (15.30+7.34 × Avg.) | b0 =[10.32 18.28], b1=[4.35 12.32] |
| **Wake After Sleep Onset (WASO, min)** | **AWS** | True | True | False | 112.68 (81.76) | 88.69 (72.79) | 23.99+8.65× Avg. | a0 =[14.40 29.73], a1=[2.91 18.26] | Bias ± 2.46 (45.83+5.91 × Avg.) | b0 =[10.10 50.01], b1=[1.72 41.64] |
|  | **WSA** | - | - | True | 12.19 (15.53) | 79.22 (63.82) | 20.78+28.54× Avg. | a0 =[60.29 43.9], a1=[-3.2 -2.33] | Bias ± 2.46 (342.21+199.79 × Avg.) | b0 =[211.66 354.06], b1=[187.93 330.36] |
|  | **Emfit** | - | - | True | 16.59 (16.66) | 69.49 (59.18) | 15.81+21.13× Avg. | a0 =[92.32 69.09], a1=[-50.05 -37.46] | Bias ± 2.46 ( 310.42+184.26× Avg.) | b0 =[196.98 323.11], b1=[171.54 297.73] |
| **Sleep Efficiency (SEFF, %)** | **AWS** | False | False | False | 73.42 (13.72) | 75.8 (15.05) | - | - | - | - |
|  | **WSA** | False | False | False | 88.17 (11.10) | 77.23 (13.77) | - | - | - | - |
|  | **Emfit** | True | False | False | 90.82 (8.00) | 79.19 (12.00) | 11.63+4.01× Avg. | a0 =[5.26 12.87], a1=[2.77 10.39] | Bias ±22.67 | Bias ±[19.97 25.37] |

**
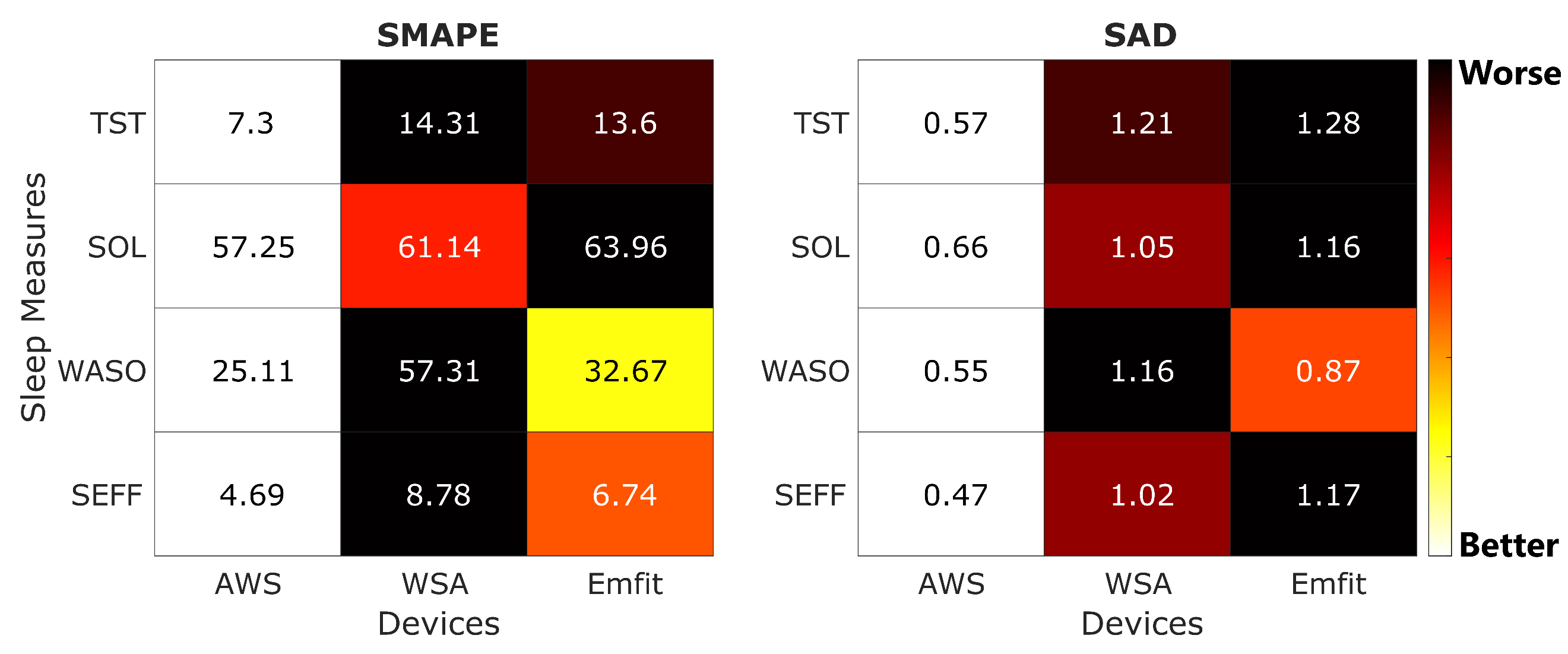
**

**Figure 5. Ranking Matrices depicting the sleep summary concordance.** The agreement matrices are represented as heatmaps to depict level of concordance with sleep diary assisted actigraphy derived metrics. For the symmetric mean absolute percentage error (SMAPE) and standardized absolute difference (SAD), each sleep measure row is colour coded using hot colormap to denote low (dark) to high (light) performance. The sleep summary measures are computed over the Analysis Period-Automatic [AP-A]. All the values are rounded to two decimal places. Unavailability of the measure is indicated using grey. The colour code of all the agreement matrices is scaled across each row.

## Choosing Actiwatch (AWS) sleep-wake threshold

The data collected during the laboratory session of the study involving 35 older adults aged between 65 and 83 (70.8±4.9 years; 14 women) was used for determining the optimal threshold setting for the AWS. The polysomnography (PSG) was recorded alongside the AWS. The PSG data were scored by two trained scores and the consensus hypnogram was generated. The hypnogram was used to generate the PSG all-night sleep summary measures.

We compared the all-night sleep summary and epoch-by-epoch concordance of Actiwatch spectrum (AWS) in AP-A (analysis performed automatically by the device software without sleep diary) and AP-SD (analysis performed with sleep diary lights off information) and three different threshold (low (L), medium (M) and high (H)) setting in the Actiwatch sleep scoring algorithm. The immobility minutes parameter was set to the default value of 10 minutes during this analysis.


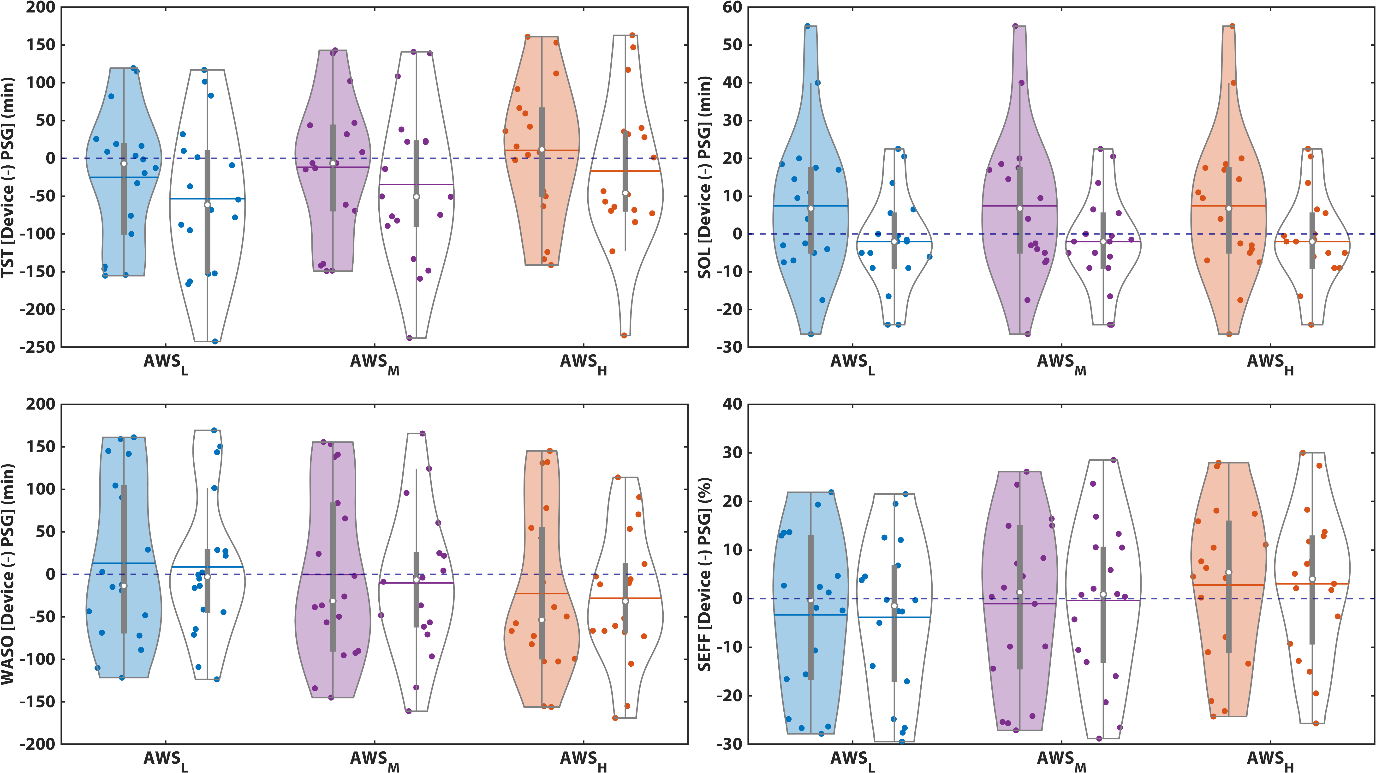
The bias of the AWS estimates was lower when the analysis period was based on the sleep diary compared to the automatic detection of the analysis period and this was true for all three sensitivity thresholds. Within each configuration (i.e., AP-SD and AP-A) the low and medium threshold provide similar agreement with polysomnography (PSG) summary and epoch-by-epoch estimates as can be seen in Tables 6 and 7. Although the estimations of total sleep time (TST), wake after sleep onset (WASO) and sleep efficiency (SEFF) varied with different setting, the sleep onset latency (SOL) estimates were identical across all AWS setting, which is in agreement with Taibi et al, 2013 *. The visualisation of differences of the different configurations against PSG is depicted in Figure 5. Since there were no performance differences between low and medium threshold settings, we used the Actiware recommended medium threshold setting for the comparison of both AP-SD and AP-A configurations of AWS with different CST.

**Figure 6.** **Differences in sleep summary estimations of AWS against PSG (AWS (-) PSG).** The number of participants used in each of the configurations are AWS AP-A [n=18] and AWS AP-SD (AWS+Sleep diary) [n=18]. The shaded violins depict the automated estimates, and the clear violins depict the lights off period estimates. The line of no deviation is depicted as a blue dotted line. The data points below the line are device underestimations and above the line are overestimations. Each violin represents the distribution of estimates. The boxplot at the centre of the violin depicts the median (circle at the centre), 1st and 3rd quartiles (lower and upper ends of the box) and the whiskers depict the 1.5 × IQR (inter quartile range). The solid-coloured line in the violins depict the mean.

* Taibi DM, Landis CA, Vitiello MV. Concordance of polysomnographic and actigraphic measurement of sleep and wake in older women with insomnia. J Clin Sleep Med. 2013 Mar 15;9(3):217-25. doi: 10.5664/jcsm.2482.

**Table 6. Sleep/wake summary measure agreement metrics of AWS AP-A and AP-SD.**

| **Sleep measure** | | **AWS AP-A (18)** | | | **AWS AP-SD (18)** | | |
| --- | --- | --- | --- | --- | --- | --- | --- |
|  |  | **L** | **M** | **H** | **L** | **M** | **H** |
| **TST**  **(min)** | **Bias (SD)** | -25.03 (87.90)  [-68.75 18.68] | -11.72 (92.81)  [-57.87 34.43] | 10.69 (91.14)  [-34.63 56.01] | -51.29 (101.10)  [-101.56 -1.01] | -35.57 (104.73)  [-87.65 16.51] | -18.68 (104.40)  [-70.60 33.24] |
|  | **p-value** | 0.24 | 0.599 | 0.625 | 0.046 | 0.167 | 0.458 |
|  | **LoA Lower bound** | -197.32  [-273.46 -121.18] | -193.62  [-274.00 -113.23] | -167.94  [-246.88 -89.00] | -249.44  [-337.00 -161.87] | -240.84  [-331.55 -150.12] | -223.31  [-313.74 -132.88] |
|  | **LoA Upper bound** | 147.25  [71.12 223.39] | 170.18  [89.8 250.57] | 189.32  [110.38 268.26] | 146.86  [59.29 234.43] | 169.70  [78.99 260.41] | 185.95  [95.52 276.38] |
|  | **MDC** | 172.29 | 181.90 | 178.63 | 198.15 | 205.27 | 204.63 |
|  | **SAD** | 0.93  [0.44 1.41] | 0.93  [0.45 1.41] | 0.95  [0.47 1.43] | 1.17  [0.68 1.65] | 1.09  [0.61 1.57] | 1.02  [0.54 1.51] |
|  | **SMAPE** | 8.91  [4.86 12.96] | 9.06  [5.17 12.95] | 8.87  [5.43 12.31] | 12.34  [7.51 17.16] | 11.75  [7.13 16.37] | 11.06  [6.73 15.39] |
|  | **ICC** | 0.32  [-0.13 0.81] | 0.32  [-0.17 0.81] | 0.34  [-0.15 0.82] | 0.18  [-0.21 0.72] | 0.21  [-0.23 0.75] | 0.27  [-0.22 0.78] |
| **SOL**  **(min)** | **Bias (SD)** | 7.44 (19.66)  [-2.33 17.22] | 7.44 (19.66)  [-2.33 17.22] | 7.44 (19.66)  [-2.33 17.22] | 7.44 (16.39)  [-0.71 15.59] | 7.44 (16.39)  [-0.71 15.59] | 7.44 (16.39)  [-0.71 15.59] |
|  | **p-value** | 0.126 | 0.126 | 0.126 | 0.07 | 0.07 | 0.07 |
|  | **LoA Lower bound** | -31.09  [-48.13 -14.06] | -31.09  [-48.13 -14.06] | -31.09  [-48.13 -14.06] | -24.68  [-38.87 -10.48] | -24.68  [-38.87 -10.48] | -24.68  [-38.87 -10.48] |
|  | **LoA Upper bound** | 45.98  [28.95 63.01] | 45.98  [28.95 63.01] | 45.98  [28.95 63.01] | 39.57  [25.37 53.76] | 39.57  [25.37 53.76] | 39.57  [25.37 53.76] |
|  | **MDC** | 38.54 | 38.54 | 38.54 | 32.12 | 32.12 | 32.12 |
|  | **SAD** | 1.18  [0.70 1.67] | 1.18  [0.70 1.67] | 1.18  [0.70 1.67] | 0.79  [0.30 1.27] | 0.79  [0.30 1.27] | 0.79  [0.30 1.27] |
|  | **SMAPE** | 59.28  [42.12 76.43] | 59.28  [42.12 76.43] | 59.28  [42.12 76.43] | 45.48  [28.13 62.83] | 45.48  [28.13 62.83] | 45.48  [28.13 62.83] |
|  | **ICC** | -0.05  [-0.45 0.56] | -0.05  [-0.45 0.56] | -0.05  [-0.45 0.56] | 0.39  [-0.03 0.83] | 0.39  [-0.03 0.83] | 0.39  [-0.03 0.83] |
| **WASO**  **(min)** | **Bias (SD)** | 13.06 (96.67)  [-35.01 61.13] | -0.25 (100.66)  [-50.31 49.8] | -22.66 (97.08)  [-70.94 25.61] | 6.93 (85.43)  [-35.55 49.41] | -8.79 (85.83)  [-51.48 33.89] | -25.68 (81.11)  [-66.01 14.66] |
|  | **p-value** | 0.574 | 0.99 | 0.335 | 0.735 | 0.669 | 0.196 |
|  | **LoA Lower bound** | -176.41  [-260.14 -92.68] | -197.55  [-284.74 -110.36] | -212.93  [-297.02 -128.85] | -160.51  [-234.5 -86.52] | -177.03  [-251.37 -102.68] | -184.65  [-254.91 -114.4] |
|  | **LoA Upper bound** | 202.53  [118.80 286.26] | 197.04  [109.85 284.23] | 167.61  [83.53 251.69] | 174.36  [100.37 248.36] | 159.44  [85.10 233.79] | 133.3  [63.04 203.55] |
|  | **MDC** | 189.47 | 197.30 | 190.27 | 167.44 | 168.23 | 158.97 |
|  | **SAD** | 1.04  [0.55 1.52] | 1.08  [0.59 1.56] | 1.14  [0.65 1.62] | 0.94  [0.45 1.42] | 0.95  [0.47 1.43] | 1.00  [0.51 1.48] |
|  | **SMAPE** | 29.20  [21.26 37.13] | 34.18  [24.95 43.42] | 41.52  [30.15 52.89] | 23.26  [14.42 32.1] | 28.04  [16.82 39.26] | 33.83  [20.45 47.2] |
|  | **ICC** | 0.26  [-0.24 0.78] | 0.24  [-0.27 0.77] | 0.25  [-0.23 0.77] | 0.26  [-0.25 0.78] | 0.27  [-0.24 0.79] | 0.31  [-0.14 0.8] |
| **SEFF**  **(%)** | **Bias (SD)** | -3.34 (16.56)  [-11.58 4.89] | -1.03 (17.33)  [-9.65 7.59] | 2.81 (16.4)  [-5.35 10.96] | -4.83 (16.58)  [-13.07 3.42] | -1.97 (16.99)  [-10.42 6.48] | 1.06 (16.52)  [-7.16 9.27] |
|  | **p-value** | 0.403 | 0.804 | 0.477 | 0.233 | 0.629 | 0.789 |
|  | **LoA Lower bound** | -35.80  [-50.14 -21.46] | -34.99  [-50 -19.98] | -29.33  [-43.53 -15.13] | -37.33  [-51.69 -22.96] | -35.26  [-49.98 -20.55] | -31.32  [-45.63 -17.01] |
|  | **LoA Upper bound** | 29.11  [14.77 43.45] | 32.94  [17.93 47.95] | 34.95  [20.74 49.15] | 27.68  [13.31 42.04] | 31.33  [16.61 46.04] | 33.44  [19.13 47.74] |
|  | **MDC** | 32.45 | 33.97 | 32.14 | 32.50 | 33.29 | 32.38 |
|  | **SAD** | 1.05  [0.57 1.54] | 1.07  [0.58 1.55] | 1.08  [0.60 1.57] | 1.02  [0.54 1.50] | 0.98  [0.50 1.47] | 0.98  [0.50 1.47] |
|  | **SMAPE** | 10.23  [6.36 14.09] | 10.44  [6.76 14.13] | 9.88  [6.70 13.06] | 10.06  [5.90 14.22] | 9.71  [5.77 13.64] | 9.44  [5.96 12.92] |
|  | **ICC** | 0.23  [-0.26 0.76] | 0.21  [-0.30 0.76] | 0.25  [-0.24 0.78] | 0.24  [-0.22 0.77] | 0.27  [-0.23 0.79] | 0.32  [-0.19 0.81] |

The number of participants used in each of the two configurations is represented adjacent to the configuration name within ‘()’. The values shown are the mean (standard deviation) followed by the 95% confidence interval. The metrics include Bias – difference in measurement between the PSG and Device (PSG – Device); Standardized difference (Std. Diff.) – Cohen’s d (effect size depicting the magnitude of differences); Minimum detectable change (MDC) – smallest detectable change independent of measurement error (half of Bland Altman agreement width); Absolute difference – difference in absolute measurement value between the PSG and Device; Standardized absolute difference (SAD) – directionless version of Cohen’s d; Symmetric mean absolute percentage error (SMAPE) – mean error in measurement; Pearson’s correlation () and Consistency intraclass correlation with two-way random effects (ICC) – measures of measurement reliability; paired t-test (t-test) – significance of differences between the device and PSG (‘-’, no significant difference, p < 0.05).


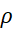

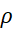

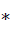

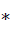


**Table 7. Sleep/Wake epoch bssssy epoch concordance measures for AWS at low (L), medium (M) and high (H) thresholds**

| **Sleep Stage** | | **Sensitivity** | **Specificity** | **Accuracy** | **MCC** | **F1 Score** |
| --- | --- | --- | --- | --- | --- | --- |
| Sleep/Wake | AWS L | 0.91 (0.03)  [0.89, 0.92] | 0.44 (0.13)  [0.38, 0.51] | 0.78 (0.05)  [0.75, 0.80] | 0.39 (0.1)  [0.35, 0.44] | 0.85 (0.05)  [0.83, 0.87] |
|  | AWS M | 0.95 (0.02)  [0.94, 0.96] | 0.34 (0.12)  [0.28, 0.40] | 0.78 (0.06)  [0.74, 0.81] | 0.37 (0.11)  [0.32, 0.43] | 0.85 (0.05)  [0.83, 0.88] |
|  | AWS H | 0.98 (0.01)  [0.97, 0.98] | 0.24 (0.11)  [0.19, 0.29] | 0.77 (0.07)  [0.73, 0.80] | 0.33 (0.11)  [0.28, 0.39] | 0.85 (0.05)  [0.83, 0.88] |

The values shown are the mean (standard deviation) followed by the 95% confidence interval.


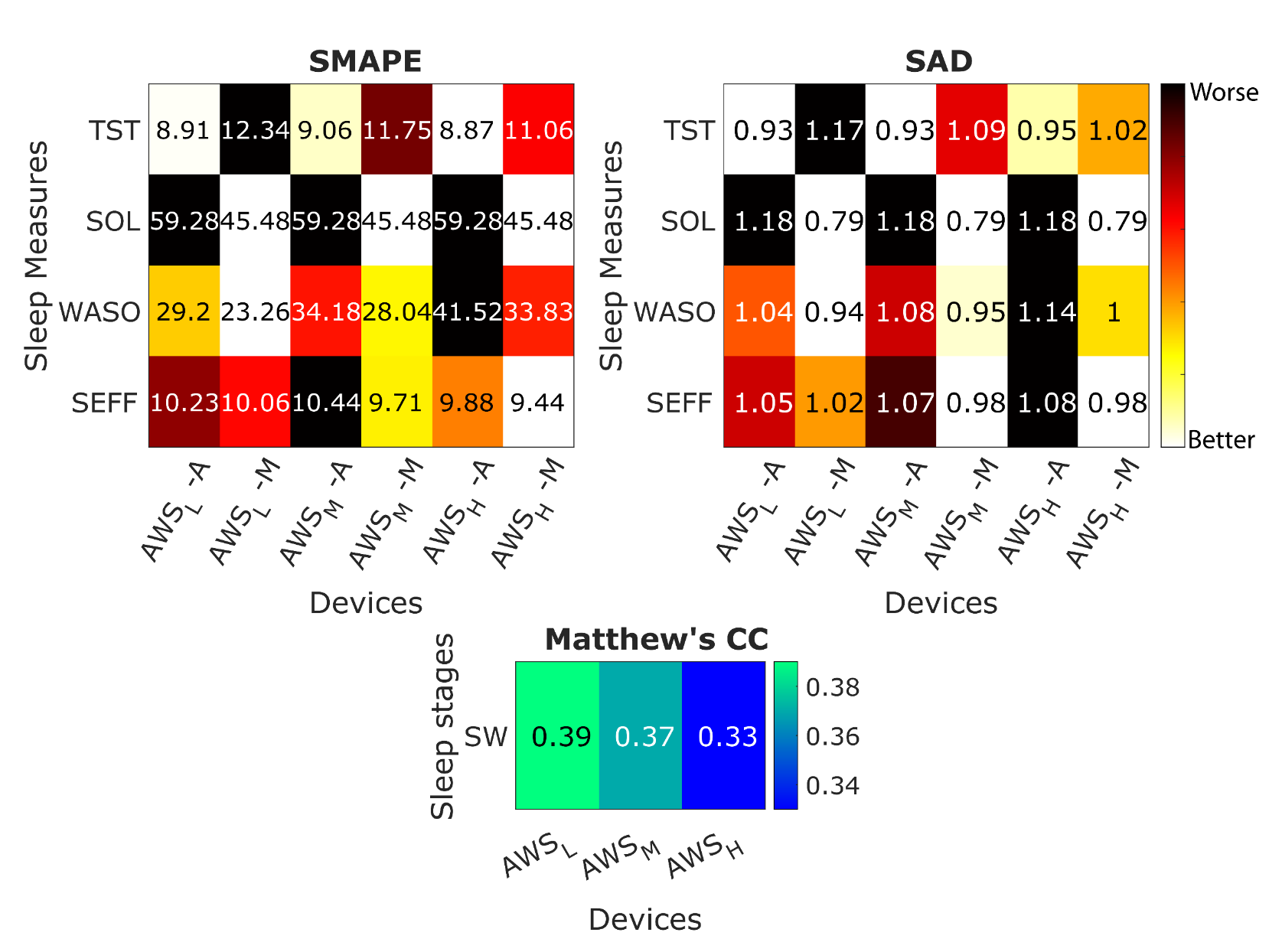


**Figure 7. Agreement Matrices depicting AWS sleep summary and EBE concordance.** The agreement matrices are represented as heatmaps to depict level of concordance with polysomnography derived metrics. For the symmetric mean absolute percentage error (SMAPE) and standardized absolute difference (SAD), each sleep measure row is colour coded using hot colormap to denote low (dark) to high (light) performance. The detailed description of the sleep summary agreement metrics is given in the sleep summary agreement assessment. For the sleep stage agreement, Matthew’s correlation coefficient (Matthew’s CC) is used.

**Figure
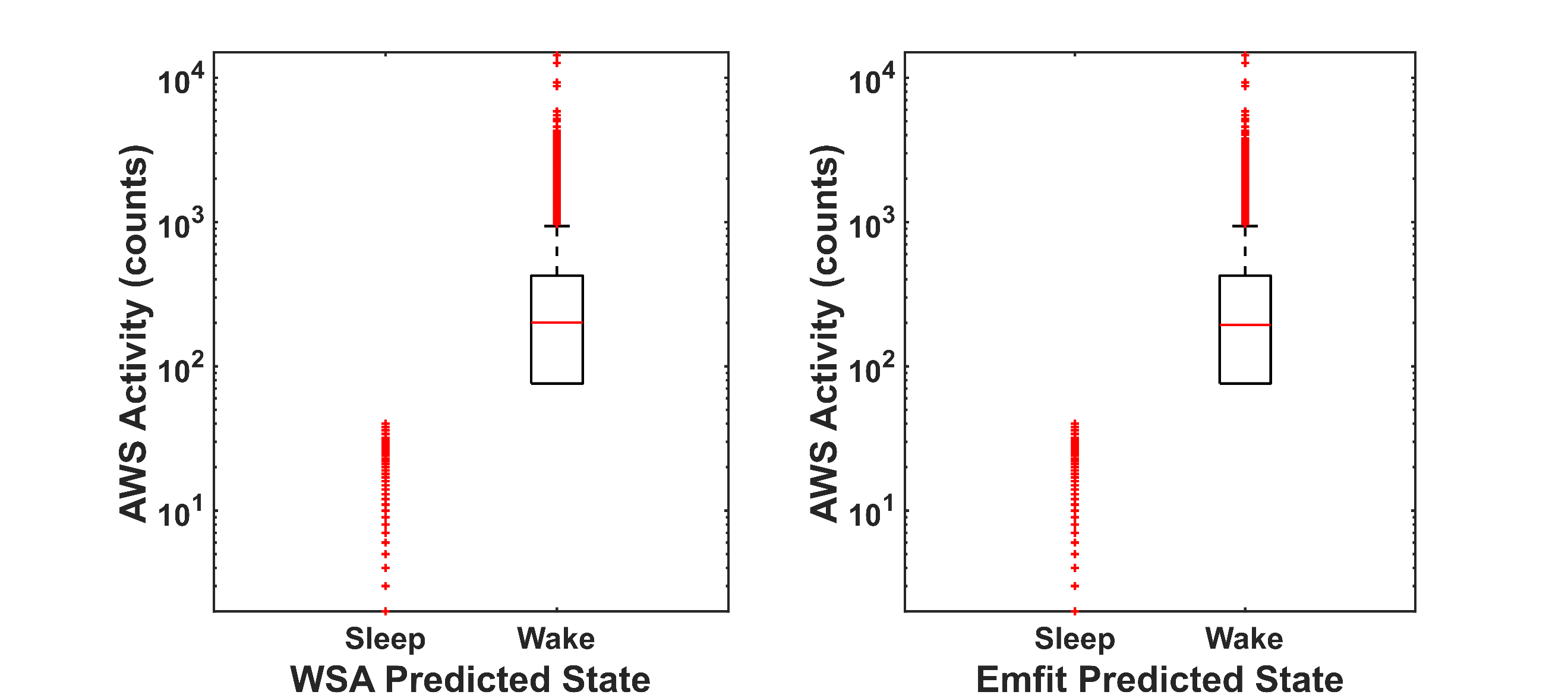
8. Activity levels during Sleep and Wake as predicted by the contactless devices. The plot on the left depicts the WSA and one on the right depicts the Emfit.**


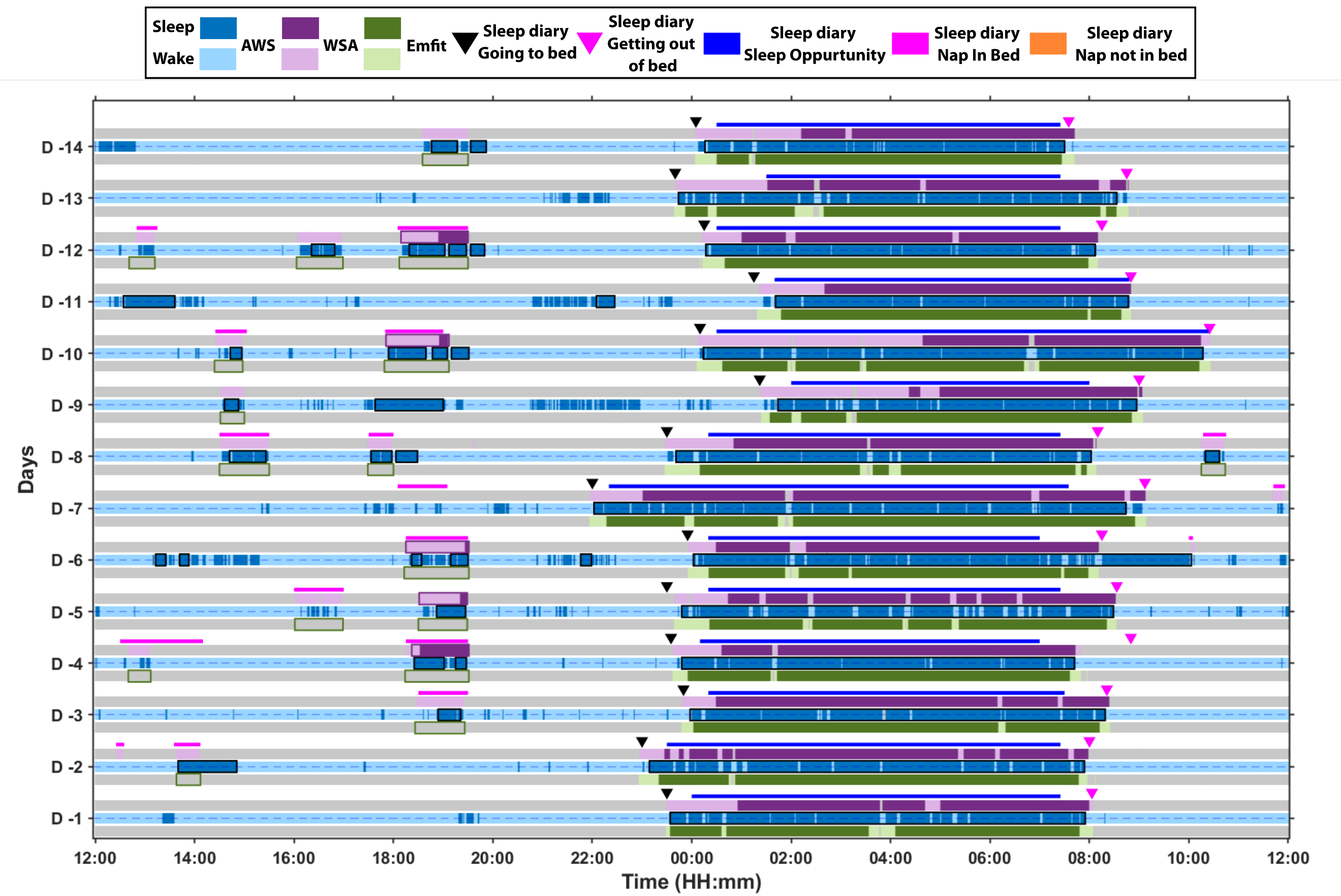
**Figure 9. Plot showing the discrepancy between the device bed occupancy information summaries during naps.** Sleep behaviour patterns over 14 days (D-14 to D-1) at home in a male participant aged 74 is shown. The short summaries or naps automatically detected by the devices are highlighted by boxes of their respective colours. Although Emfit epoch by epoch data was unavailable, the automatic short summaries coincided with the sleep diary summaries. The WSA detected in bed periods (region coloured in purple without a box) accurately for all naps in bed but automatic summaries were generated only when the device determined that participant had sleep epochs in the in-bed period.
